# Spatiotemporal spread of tick-borne encephalitis in the EU/EEA, 2012 to 2020

**DOI:** 10.2807/1560-7917.ES.2023.28.11.2200543

**Published:** 2023-03-16

**Authors:** Jasper Van Heuverswyn, Luisa K. Hallmaier-Wacker, Julien Beauté, Joana Gomes Dias, Joana M Haussig, Kristina Busch, Jana Kerlik, Mateusz Markowicz, Henna Mäkelä, Teresa Marie Nygren, Hana Orlíková, Maja Socan, Jakub Zbrzeźniak, Milda Žygutiene, Céline M Gossner

**Affiliations:** 1 European Centre for Disease Prevention and Control (ECDC), Stockholm, Sweden; 2 Public Health Agency of Sweden, Stockholm, Sweden; 3 Regional Authority of Public Health in Banská Bystrica, Banská Bystrica, Slovakia; 4 Austrian Agency for Health and Food Safety, Vienna, Austria; 5 Finnish Institute for Health and Welfare, Helsinki, Finland; 6 Robert Koch Institute (RKI), Berlin, Germany; 7 National Institute of Public Health, Prague, Czechia; 8 National Institute of Public Health, Ljubljana, Slovenia; 9 National Institute of Public Health - NIH - National Research Institute, Warsaw, Poland; 10 National Public Health Center under the Ministry of Health, Vilnius, Lithuania

**Keywords:** Tick-borne encephalitis, European Union, surveillance, epidemiology, vaccination, public health

## Abstract

**Background:**

Tick-borne encephalitis (TBE) is a vaccine-preventable disease involving the central nervous system. TBE became a notifiable disease on the EU/EEA level in 2012.

**Aim:**

We aimed to provide an updated epidemiological assessment of TBE in the EU/EEA, focusing on spatiotemporal changes.

**Methods:**

We performed a descriptive analysis of case characteristics, time and location using data of human TBE cases reported by EU/EEA countries to the European Centre for Disease Prevention and Control with disease onset in 2012–2020. We analysed data at EU/EEA, national, and subnational levels and calculated notification rates using Eurostat population data. Regression models were used for temporal analysis.

**Results:**

From 2012 to 2020, 19 countries reported 29,974 TBE cases, of which 24,629 (98.6%) were autochthonous. Czechia, Germany and Lithuania reported 52.9% of all cases. The highest notification rates were recorded in Lithuania, Latvia, and Estonia (16.2, 9.5 and 7.5 cases/100,000 population, respectively). Fifty regions from 10 countries, had a notification rate ≥ 5/100,000. There was an increasing trend in number of cases during the study period with an estimated 0.053 additional TBE cases every week. In 2020, 11.5% more TBE cases were reported than predicted based on data from 2016 to 2019. A geographical spread of cases was observed, particularly in regions situated north-west of known endemic regions.

**Conclusion:**

A close monitoring of ongoing changes to the TBE epidemiological situation in Europe can support the timely adaption of vaccination recommendations. Further analyses to identify populations and geographical areas where vaccination programmes can be of benefit are needed.

**Key public health message**

What did you want to address in this study?

Tick-borne encephalitis (TBE) is a vaccine-preventable disease which affects the central nervous system. The causal virus is found in many European countries but in recent years, several regions have detected their first human TBE cases or reported an increase in cases. As a result, it is necessary to update the epidemiological assessment of TBE in Europe with a focus on where and at what time of the year cases occur.

What have we learnt from this study?

TBE cases increased between 2012 and 2020, and there was a north-west bound spread in continental Europe. Using the data reported to the central notification system run by the European Union, we were able to create updated geographical maps and notification rates at subnational level for 17 countries in Europe.

What are the implications of your findings for public health?

The results can support updated vaccination and health promotion campaigns in areas with high numbers of TBE cases. The results should also help to increase awareness among medical practitioners and highlight areas where increased surveillance is warranted. They show the strengths of the European TBE surveillance network and identify some shortcomings. Strengthened cooperation through data and expertise sharing is necessary.

## Introduction

Tick-borne encephalitis (TBE) is a vaccine-preventable disease caused by the tick-borne encephalitis virus (TBEV), which is found in most of Europe and northern Asia. The virus belongs to the genus *Flavivirus* and consists of three main subtypes: the European (TBEV-Eu), the Siberian (TBEV-Sib) and the Far Eastern (TBEV-FE). In the European Union/European Economic Area (EU/EEA), TBEV-Eu is the predominant subtype; cocirculation of all three subtypes has however been reported in the Baltic countries Estonia, Latvia and Lithuania [1].

TBEV mainly circulates among ticks (*Ixodes ricinus* for TBEV-Eu; *Ixodes persulcatus* for TBEV-Sib and TBEV-FE) and small mammals, the latter amplifying the tick population and maintaining viral transmission [1]. Larger mammals, including humans, can become infected through bites of infected ticks but are dead-end hosts. Less common modes of transmission have also been described such as the consumption of unpasteurized milk products from infected livestock [2].

The incubation period for TBE in humans is usually 2 weeks (range 2–28 days) [3]. It has been estimated that of those infected with TBEV more than 70% remain asymptomatic, regardless of the subtype [3]. Symptomatic patients infected with TBEV-Eu typically present with a biphasic disease with a short period of recovery in between. The first phase is characterised by a non-specific influenza-like illness while the second phase features neurologic manifestations such as meningitis or encephalitis [3]. Up to 50% of cases with encephalitis develop long-term neurological and neuropsychiatric sequelae [3]. Infections with TBEV-Sib and TBEV-FE are predominantly monophasic. The case fatality rate of TBE is estimated to be less than 1% for the European and Siberian subtype but can be as high as 40% for the Far Eastern subtype [3,4]. There is no specific treatment available for TBE, but the disease can be prevented through vaccination.

TBE became a notifiable disease at the EU level in 2012. A descriptive analysis based on 2012–2016 data was published, highlighting the regions with the highest notification rates and the absence of an apparent temporal trend [5]. Since then we have observed signals indicating geographical spread of human TBE cases and an increase in number of cases reported.

In this study, we aim to provide an updated analysis of the epidemiological situation of TBE in the EU/EEA, with a focus on geographical and temporal changes since 2012. This analysis may provide useful information for the review and refinement of risk areas and prevention policies.

## Methods

### Data source, setting and study population

European Union/European Economic Area countries report TBE data to the European Centre for Disease Prevention and Control (ECDC) on a yearly basis. The surveillance system has been described elsewhere [6]. All TBE data were extracted from The European Surveillance System (TESSy). Data on population counts were extracted from Eurostat [7]. We included all TBE cases reported to ECDC by EU/EEA countries with onset of disease between 1 January 2012 and 31 December 2020. Only countries that provided yearly case-based data have been included, even if there were zero cases to be reported. Excluded countries are listed in the Supplementary Figure S1, a study flowchart representing inclusion and exclusion of cases. Cases with unknown importation status were excluded.

### Variables, and definitions

Variables used for the description of case characteristics were principally used as reported.

European Union/ European Economic Area countries are requested to report TBE cases according to the EU case definition for TBE, which has remained unchanged since its adoption in 2012 [8]. Based on the number of received TBE vaccine doses, cases are classified as fully, partially or not vaccinated. Full vaccination was defined as having received at least the three doses required for primary immunisation, with or without booster doses [4,9]. Partial vaccination was defined as having received one or two doses for primary immunisation.

Cases were classified as autochthonous or imported, as reported. If the importation status was unknown, the variable was completed based on the concordance between the reporting country and the country of infection. In agreement with the Finnish Institute for Health and Welfare, 318 cases reported by Finland with missing importation status and place of infection were classified as autochthonous. Spatial analyses were performed at EU/EEA level, country level and subnational level. The second and third level of the Nomenclature of territorial units for statistics (NUTS) were used for subnational analysis, depending on availability [10]. Place of infection was the primary variable of interest for all cases. Whenever the place of infection was not available for autochthonous cases, place of residence and place of notification were taken as proxies (in respective order, depending on availability). The concordance between the different variables is presented in Supplementary Tables S1–S3. To minimise bias, only NUTS regions with verified TBE transmission were eligible as a proxy. Verified TBE transmission was based on place of infection in the TESSy database and the scientific literature. Retained NUTS regions based on scientific literature are shown in the Supplement S3.

Temporal analyses were based on the date of disease onset. Whenever this variable was unavailable, the earliest date from statistics date, diagnosis date or notification date was taken as a proxy. The date used for statistics is the only mandatory date to be reported. It can refer to any date between the date of infection and the date of reporting, as chosen by the reporter. The disease onset date or its proxy are referred to as disease onset date throughout the manuscript. For evaluation of north-south differences in seasonality, countries were divided as follows: Estonia, Finland, Latvia, Lithuania, Norway and Sweden were classified as northern countries while the remaining countries were classified as central/southern countries.

### Analysis

We performed a descriptive analysis of case characteristics, time, and place stratified by importation status. Mann–Whitney U-tests (MWU) and Pearson’s chi-squared tests were used respectively for comparisons between continuous and categorical variables. All tests were performed two-sided with a significance level of 0.05.

Linear regression modelling was used to evaluate temporal trends at EU/EEA and country level. Date of disease onset by calendar week was used as time variable. Models were adjusted for seasonality by introducing Fourier terms. A harmonic regression model with Fourier terms and autoregressive integrated moving average (ARIMA) errors [11] was used to retrospectively predict TBE cases in 2020 based on 2016–2019 data. Comparison between the observed and predicted values allowed us to evaluate the percentual change in TBE cases in 2020.

Notification rates are presented per 100,000 population and calculated according to the following formula:


$${Notification\;rate}_{year}=\;\frac{{Number\;autochthonous\;cases}_{year}}{{Population\;count\;on\;1\;January}_{year}}\;x\;100,000$$

The mean notification rate over the entire study period was calculated by dividing the sum of the cases by the sum of population counts.

All analysis was performed using R software version 4.0.2, including the packages tidyverse, tsibble and fable [11]. Maps were produced with the ECDC Map Maker tool (https://www.ecdc.europa.eu/en/publications-data/ecdc-map-maker-tool-emma).

## Results

### Overall case characteristics

Nineteen countries reported 24,974 TBE cases between 2012 and 2020. A more detailed study flowchart is shown in the Supplementary Figure S1. Cases were predominantly autochthonous (98.6%), male (59.5%) and confirmed (93.2%). The median age of the cases was 49 years (IQR: 33–62). Most cases were hospitalised (19,700, 78.9%); there were 93 deaths due to TBE (case fatality rate = 0.4%) and 5.4% (1,347) of all cases had long-term sequelae (Table 1).

**Table 1:** Characteristics of tick-borne encephalitis cases, EU/EEA countries, 2012–2022 (n = 29,974)

Characteristics	Cases					
	Autochthonous (n = 24,629)		Imported (n = 345)		Overall (n = 24,974)	
	n	%	n	%	n	%
Sex						
Female	10,010	40.6	104	30.1	10,114	40.5
Male	14,614	59.3	241	69.9	14,855	59.5
Unknown	5	0	0	0	5	0
Age (years)						
Mean (SD)	46.8	-19.8	45.1	-18.3	46.8	-19.8
Median (IQR)	49	(33.0–62.0)	46	(32.0–59.0)	49	(33.0–62.0)
Classification						
Confirmed	22,959	93.2	326	94.5	23,285	93.2
Probable	1,249	5.1	9	2.6	1,258	5
Unknown	421	1.7	10	2.9	431	1.7
Hospitalisation						
No	1,731	7	23	6.7	1,754	7
Yes	19,451	79	249	72.2	19,700	78.9
Unknown	3,447	14	73	21.2	3,520	14.1
Outcome						
Alive	18,520	75.2	253	73.3	18,773	75.2
Alive with sequelae	1,346	5.5	1	0.3	1,347	5.4
Death due to TBE	93	0.4	0	0	93	0.4
Death due to other cause	1	0	0	0	1	0
Unknown	4,669	19	91	26.4	4,760	19.1
TBE vaccination status						
Not vaccinated	17,774	72.2	253	73.3	18,027	72.2
Partially vaccinated	505	2.1	11	3.2	516	2.1
Fully vaccinated	401	1.6	2	0.6	403	1.6
Unknown	5,949	24.2	79	22.9	6,028	24.1

EU/EEA: European Union/European Economic Area; IQR: Interquartile range; SD: standard deviation; TBE: tick-borne encephalitis.

A total of 403 (1.6%) cases were fully vaccinated. The majority of those (n = 362) were reported by Sweden (n = 157), Germany (n = 121), Czechia (n = 47) and Austria (n = 37). Vaccinated cases were older than unvaccinated cases (median age vaccinated cases: 57.0 years, median age unvaccinated cases: 49.0 years, MWU, p < 0.001). Among the fully vaccinated with a known date of last vaccine dose (332 cases), 72.6% received their last vaccine dose within 5 years before the infection and 89.5% within 10 years. A total of 516 cases were partially vaccinated (Table 1).

### Geographical analysis of autochthonous cases

Nineteen EU/EEA countries reported yearly case-based data to ECDC during the study period (Table 2). The highest number of autochthonous cases were reported by Czechia (n = 5,522), Lithuania (n = 4,196) and Germany (n = 3,309), representing 52.9% of all autochthonous cases reported. Ireland and Spain did not report any autochthonous cases (Table 2). The highest mean notification rates were recorded in the three Baltic countries: Lithuania with 16.2 cases per 100,000 population, followed by Latvia (9.5) and Estonia (7.5) (Table 2). In 2020, Lithuania had a yearly notification rate of 24.3 cases per 100,000 population (Table 2), which was the highest notification rate recorded at country level during the study period. The mean notification rate for all included countries was 0.8 cases per 100,000 population with a peak of 1.0 in 2020 (Table 2).

**Table 2:** Yearly notification rate of autochthonous tick-borne encephalitis cases per 100,000 population, EU/EEA countries, 2012–2020

Country	2012	2013	2014	2015	2016	2017	2018	2019	2020	Total
Austria	0.57	1.16	0.89	0.77	1.02	1.36	1.89	1.15	2.67	1.29
Czechia	5.44	5.91	3.89	3.28	5.29	6.48	6.74	7.27	7.87	5.80
Estonia	13.43	8.64	6.31	8.67	6.16	6.54	6.29	6.27	5.19	7.50
Finland	0.72	0.70	0.86	1.24	1.11	1.54	1.43	1.25	1.65	1.17
France	0	0	0.01	0.01	0.03	0	0.03	0	0.06	0.02
Germany	0.23	0.50	0.29	0.24	0.38	0.55	0.65	0.44	0.75	0.45
Greece	0	0	0.01	0.01	0	0	0	0	0	0
Hungary	0.44	0.51	0.31	0.24	0.19	0.15	0.32	0.18	0.18	0.28
Ireland	0	0	0	0	0	0	0	0	0	0
Latvia	11.15	11.41	7.39	7.15	10.36	11.08	7.08	10.73	9.38	9.53
Lithuania	12.52	13.83	11.86	11.33	21.81	16.33	13.53	20.58	24.30	16.15
The Netherlands	0	0	0	0	0.01	0.01	0.01	0.01	0.03	0.01
Norway	0.12	0.08	0.20	0.15	0.17	0.21	0.42	0.53	0.67	0.29
Poland	0.49	0.59	0.51	0.40	0.75	0.76	0.54	0.68	0.38	0.57
Romania	0.01	0.01	0.01	0	0	0	0.01	0	0	0.01
Slovakia	1.87	3.01	2.12	1.55	3.15	1.40	2.88	2.90	3.37	2.47
Slovenia	7.98	14.91	4.85	3.01	4.02	4.94	7.40	5.33	8.92	6.82
Spain	0	0	0	0	0	0	0	0	0	0
Sweden	2.98	2.15	1.82	2.65	2.36	3.83	3.7	3.41	2.54	2.84
**EU/EEA**	**0.70**	**0.83**	**0.57**	**0.53**	**0.79**	**0.85**	**0.88**	**0.88**	**1.02**	**0.78**

EU/EEA: the total of the presented European Union/European Economic Area countries.

**Figure 1:**
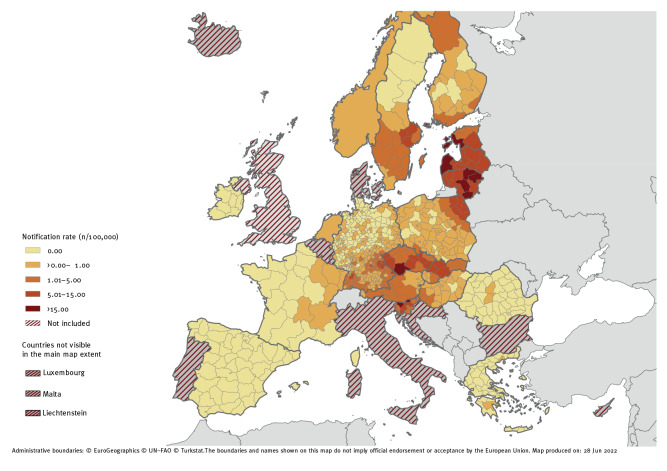
Notification rates of autochthonous tick-borne encephalitis cases per 100,000 population, EU/EEA countries, 2012–2020

For subnational spatial analyses, NUTS-3 regions were available in fifteen countries. Austria and France reported information at NUTS-2 region while the Netherlands and Norway only provided country level data. Fifty-three autochthonous cases did not have information at subnational level and were therefore not included in the subnational analysis. More details on the regional spatial analysis can be found in the Supplementary Table S4. A total of 50 regions had a mean notification rate equal to or above 5.0 per 100,000 population. These were located in Czechia, Estonia, Finland, Germany, Latvia, Lithuania, Poland, Sweden, Slovakia, and Slovenia. The highest notification rates were found in the Baltic countries Estonia, Latvia, and Lithuania followed by Czechia, Slovenia and Finland (Figure 1). All six countries had at least one NUTS-3 region with a mean notification rate above 15.0 per 100,000 population. The highest mean and yearly notification rate was recorded in Utena county (Lithuania) with 46.4 cases per 100,000 population and 62.4 cases per 100,000 population in 2020. Notification rates per year and per region can be found in the Supplementary Table S5.

NUTS: Nomenclature of territorial units for statistics [10].

NUTS-3 levels are shown whenever available. France and Austria are shown in NUTS-2 levels. Norway and the Netherlands are shown at country level.

**Figure 2:**
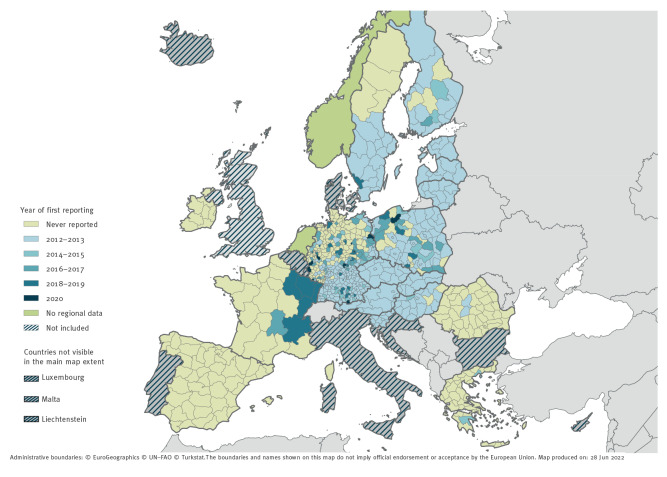
Tick-borne encephalitis cases by year of first reporting and NUTS levels, EU/EEA countries, 2012–2020

Out of the 786 included NUTS-2 and NUTS-3 regions, there were 361 regions (45.9%) where no TBE cases were reported during the study period. The notification rates per year and per regions are provided in Supplementary Table S5. For 69.4% (n = 295) of NUTS-2 and NUTS-3 regions with reported cases, TBE cases were already reported in 2012–2013. Austria, Czechia, Estonia, Latvia, Lithuania, Slovakia and Slovenia reported TBE cases in all their regions in 2012–2013. In 2012–2013, Hungary reported TBE cases in > 80% of its regions, while Finland, Poland, and Sweden reported cases in > 50% of their regions. In Germany in 2012–2013, TBE cases were reported in 34.9% (n = 140) of NUTS-3 regions. Since 2014, an additional 130 NUTS-2 and NUTS-3 regions (30.6% of regions with reported cases) reported TBE cases in Germany (n = 99), Poland (n = 18), France (n = 6), Finland (n = 3), Greece (n = 2), Hungary (n = 1) and Sweden (n = 1) (Figure 2).

NUTS: Nomenclature of territorial units for statistics [10].

Austria and France are shown at NUTS-2 level. Other countries are presented at NUTS-3 level.

### Temporal analysis of autochthonous cases

**Figure 3:**
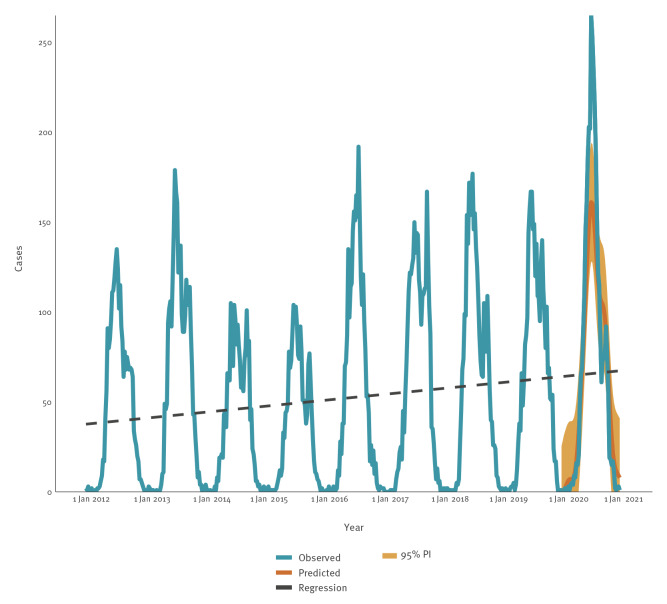
Number of autochthonous tick-borne encephalitis cases reported by week for EU/EEA countries with complete reporting, 2012–2020 (n = 24,629)

TBE transmission occurred year-round, but most cases (98.8%, 24,679/24,974) had a disease onset between April and November. In all years except for 2012 and 2016, a bimodal distribution of autochthonous cases was observed. The first peak occurred around the first week of July, with a second (usually smaller) peak at the end of September (Figure 3). Accordingly, July was most frequently reported as the month of disease onset. In northern countries, the main peak was observed later (July–August) compared with central/southern countries (June–July) (MWU, p < 0.05). In addition, the transmission level remained high in the northern countries throughout summer with a decrease from October, while there was already a substantial decrease in cases in August for the central/southern countries.

EU/EEA: European Union/European Economic Area; PI: prediction interval.

Dashed line presents result of linear regression. Red line presents predicted values for 2020 based on 2016–2019 data.

The 19 EU/EEA countries include: Austria, Czechia, Estonia, Finland, France, Germany, Greece, Hungary, Ireland, Latvia, Lithuania, The Netherlands, Norway, Poland, Romania, Slovakia, Slovenia, Spain, Sweden.

The epidemiology of TBE cases in the EU/EEA was characterised by annual variation with an increasing trend in the last 6 years of the study. The lowest number of cases and notification rate were registered in 2015 (1,857 cases, 0.5 cases per 100,000 population). Since then, there has been a gradual increase with the highest number of cases (3,604) reported in 2020, corresponding to a notification rate of 1.0 cases per 100,000 population (Table 2, Table 3).

**Table 3:** Number of autochthonous tick-borne encephalitis cases by year^a^ and trend analysis^b^ in EU/EEA countries, 2012–2020

Country	Cases per year									Total number of cases	Trend analysis		
	2012	2013	2014	2015	2016	2017	2018	2019	2020		Coefficient^c^	95% CI	p value
Austria	48	98	76	66	89	119	167	102	238	1,003	0.006	0.005 to 0.008	< 0.001
Czechia	571	621	409	346	558	686	715	774	842	5,522	0.015	0.011 to 0.020	< 0.001
Estonia	178	114	83	114	81	86	83	83	69	891	-0.003	-0.005 to -0.002	< 0.001
Finland	39	38	47	68	61	85	79	69	91	577	0.002	0.002 to 0.003	< 0.001
France	0	1	7	7	18	1	20	2	43	99	0.001	0.001 to 0.002	< 0.001
Germany	185	400	232	195	314	451	539	367	626	3,309	0.016	0.012 to 0.019	< 0.001
Greece	0	0	1	1	0	0	0	0	0	2	NA	NA	NA
Hungary	44	51	31	24	19	15	31	18	18	251	-0.001	-0.002 to -0.001	< 0.001
Ireland	0	0	0	0	0	0	0	0	0	0	NA	NA	NA
Latvia	228	231	148	142	204	216	137	206	179	1,691	-0.001	-0.003 to 0.000	0.105
Lithuania	376	411	349	331	630	465	380	575	679	4,196	0.012	0.007 to 0.016	< 0.001
The Netherlands	0	0	0	0	2	1	2	2	5	12	NA	NA	NA
Norway	6	4	10	8	9	11	22	28	36	134	0.001	0.001 to 0.002	< 0.001
Poland	186	225	192	151	284	287	206	257	145	1,933	0.001	-0.002 to 0.003	0.587
Romania	3	3	1	0	0	0	2	0	0	9	NA	NA	NA
Slovakia	101	163	115	84	171	76	157	158	184	1,209	0.002	0.001 to 0.004	0.007
Slovenia	164	307	100	62	83	102	153	111	187	1,269	-0.002	-0.004 to 0.000	0.037
Spain	0	0	0	0	0	0	0	0	0	0	NA	NA	NA
Sweden	283	205	176	258	232	383	374	349	262	2,522	0.005	0.003 to 0.007	< 0.001
**EU/EEA**	**2,412**	**2,872**	**1,977**	**1,857**	**2,755**	**2,984**	**3,067**	**3,101**	**3,604**	**24,629**	**0.053**	**0.040 to 0.067**	**< 0.001**

EU/EEA: total from the presented European Union/ European Economic Area countries; NA: not applicable (shown whenever the number of cases was insufficient for trend analysis).

^a^ Number of autochthonous cases per year are shown for countries with yearly reported case-based data.

^b^ Trend analysis was performed by using linear regression models with Fourier terms to adjust for seasonality.

^c^ The coefficient represents the average weekly change during the study period and is shown with 95% confidence intervals and p values.

Results of the seasonal-adjusted linear regression models suggest that there has been a statistically significant increase in TBE cases during the study period of on average of 0.053 additional TBE cases per week (95% CI: 0.040 to 0.067, p < 0.001, Table 2, Figure 3). While there was an average decrease of -0.095 cases per week (95% CI: -0.128 to -0.061, p < 0.001) during the 2012–2015 period, there was a statistically significant average increase of 0.066 additional cases per week (95% CI: 0.034 to 0.099, p < 0.001) during the 2016–2020 period. An increase was observed in most countries during the study period, with the exception of Estonia, Hungary and Slovenia which observed a decrease and Latvia and Poland which observed no change (Table 3).

In 2020, 11.5% excess TBE cases were reported compared with retrospective predictions based on 2016–2019 data (3,604 observed cases compared with 3,231 predicted). The observed increase was, however, not equally distributed throughout the year. In June and July, the case numbers exceeded the maxima of previous years and were higher than the upper limit of the 95% prediction interval (Figure 3). From August onwards, case numbers were similar or even lower than predicted.

### Imported cases

From 2012 to 2020, there were 345 imported TBE cases. Compared with autochthonous cases, imported cases were slightly younger (MWU, p-value < 0.05) and were more often male (69.9%, chi-squared test, p< 0.05) (Table 1). Most of the imported cases were infected in another EU/EEA country (81.4%), principally in Austria, Germany and Sweden. Among the 33 imported cases with a country of infection outside the EU/EEA, 17 were reported to be infected in Switzerland and four in Russia. Imported TBE cases followed the seasonal pattern of the autochthonous cases with July as the most reported month of onset. The number of imported TBE cases gradually increased from 2012 to 2018 (from 22 to 66 TBE cases, respectively). In 2019, there was a small decrease (58 cases), followed by a more distinct drop in 2020 (29 cases).

## Discussion

After analysing data from TESSy, we were able to provide updates on the temporal and geographical distribution of TBE cases in 19 EU/EEA countries and describe the demographical characteristics and vaccination status of these TBE cases.

The age and sex distribution of the presented data are in line with previously published research [3,12]. A contributing factor to the higher proportion of men could be that men and women engage differently in protective measures against tick bites [13,14]. Sex differences in occupation and leisure activities leading to a differential exposure to ticks and biological factors could possibly also contribute to this finding. Interestingly, we noted a higher male-to-female ratio among imported cases. Even though surveillance bias cannot be excluded, we hypothesise that sex differences in risk perception, with less protective behaviour in males, might be more pronounced in travellers.

Our data confirm the relatively low mortality among TBE cases in Europe [3]. The proportion of long-term sequelae, however, is much lower than has previously been reported. It is likely that this is a bias in our surveillance data since cases are not followed up after initial reporting.

The World Health Organization (WHO) recommends that TBE vaccination should be offered to all inhabitants of regions with a notification rate ≥ 5 per 100,000 population (i.e. high-endemic regions) [15]. We identified 50 regions in the EU/EEA that fulfil this criterion, and, in line with the WHO recommendation, TBE vaccination is recommended by the national authorities in most of these regions. Information on the vaccination recommendations in high endemic regions and cost coverage is provided in the Supplementary Table S6. However, a recent study has shown that the average self-reported TBE vaccination rate in many of the highly endemic regions and/or countries is low [9]. Among countries with an overall notification rate ≥ 5 of 100,000 population, Latvia has the highest vaccination rate at 52.5%, while in Czechia, Estonia, Lithuania and Slovenia vaccination rates range between 23.0% and 30.4% [9]. In contrast, with 82% of inhabitants having received at least one vaccine dose by 2018 [12,16], Austria has the highest vaccination rate among the EU/EEA countries. Since the end of the 1970s, Austria has been conducting intense awareness campaigns as well as mass vaccination campaigns against TBE; the vaccine is offered at a reduced cost during the first 6 months of the year and is free for people with an occupational risk of infection [17]. As a result, the TBE notification rate declined from 5.7 per 100,000 inhabitants for the period 1972–1982 to 1.3 for the period 2012–2020 [12].

Offering the vaccine at a reduced cost or even free of charge has been demonstrated to substantially increase the willingness to be vaccinated [18]. Studies conducted in Stockholm County, Sweden and in Slovenia showed that free TBE vaccination programmes can be cost-effective in specific age groups and even cost saving when taking into account indirect costs [19,20]. Another study conducted in Stockholm County, from the same period as the study mentioned above, came to a different conclusion; authors found it not cost effective to offer a general vaccination against TBE for the different age groups studied [21]. This contradiction highlights the complexity of performing comprehensive cost-effectiveness studies and the importance of carefully selecting the parameters feeding the models. Further cost-effectiveness studies are needed in high endemic areas to refine and adjust the vaccination strategies.

Over 400 cases in our study (1.6%) had received at least three priming doses and were thus classified as fully vaccinated. These data should be interpreted with caution as we could not assess whether the vaccination regimen of these cases followed the requirements (e.g. interval between doses) and we did not have information on possible underlying conditions that could explain a reduced protection from the vaccine. It should be noted that the collection of information from severe TBE cases is known to be challenging and collection of vaccination status from relatives might not be accurate. We observed that fully vaccinated cases were older than non-vaccinated cases despite the vaccine having been shown to be highly effective in older age groups [12]. In addition, we found that most of the fully vaccinated cases received their last dose within the past 10 years. Even though standard vaccination schedules require boosters at 3–5 year intervals [4], it has been suggested that vaccine protection remains over a 10-year period [22]. The development of a standardised laboratory protocol for the confirmation of TBE vaccine breakthrough infections and the systematic collection of these events at the EU/EEA level could provide a better understanding of this phenomenon.

Throughout the study period, 130 regions (i.e. 30.6% of regions with reported cases) were considered as newly affected when compared with 2012–2013, predominantly in France, Germany, and Poland. In this study we demonstrated that the expansion is primarily northwards and westwards. This expansion has been previously described in recent scientific literature. While countries such as Austria, Czechia and Switzerland have been considered endemic for decades [1], the first human TBE cases were reported in Belgium, the Netherlands and the United Kingdom (UK) between 2016 and 2020 [23-25]. Furthermore, countries with known endemic regions such as Denmark, France and Germany have been reporting newly affected regions north and west of their traditional foci [26-28].

Even though the main vector of TBEV in Europe, *I. ricinus*, is established in almost all regions within the EU/EEA [29], no human TBE cases have been reported in 45.9% of the included regions. The tick-borne encephalitis virus can be introduced to new areas via viraemic hosts or through the transport of infected ticks by birds or larger mammals, but the establishment of an enzootic cycle requires favourable environmental and/or climatological conditions, which are not met in all regions where the vector is established [30]. Conversely, TBEV may circulate in regions where no human cases have been reported to date, as exemplified by a serological survey performed in Denmark among roe deer that highlighted virus circulation in areas where no human cases were ever reported [28,31].

Since we did not have information on the region of infection of cases reported by Norway and the Netherlands, the distribution of human TBE cases by region is not shown in these countries. Based on the literature, we can, however, conclude that the virus circulates principally in the southernmost regions of Norway, adjacent to the coast [32], and in some focal areas in the east-central parts of the Netherlands [24].

As we aimed to describe the epidemiological situation in the EU/EEA, we herewith complement our data with a brief description of the epidemiology in EU/EEA countries that were not included in the study. Bulgaria has a low incidence with only a few TBE cases reported [33]. Similarly, Denmark and Liechtenstein only reported a few cases every year (15 and 8 cases during the study period, respectively) [28,34]. In contrast, Croatia and Italy both have between 10 and 55 TBE cases per year, all reported in the northernmost regions [35-37]. In Belgium, two possible autochthonous cases were identified in 2018 and the first confirmed cases were documented in 2020 [25,38]. For the UK, the first probable TBE case was documented in 2019 [23]. To the best of our knowledge, no autochthonous TBE cases have been documented in Cyprus, Iceland, Luxembourg, Malta, or Portugal.

Despite yearly variation, there was a significant increase in TBE cases in the EU/EEA from 2012 to 2020, particularly in the last 5 years. The drivers for long-term trends in TBE epidemiology are multifactorial; virus evolution, changes in vector and host abundance as well as human behaviour play an important role. In addition, changes in surveillance systems, diagnostic methods and vaccination policies can also have an effect on the observed trend. Most likely, our results are shaped by the intricate relationship between several of these factors with differential impacts in varying regions. This could also explain diverging trends among countries.

Distribution of cases by month of reporting differed between northern and central/southern countries. It is likely that favourable environmental conditions for an abundance of small mammals and tick activity occurs earlier in the year in central/southern countries. However, we do not think that the timing of human activities associated with a high risk of infection (e.g. mushroom picking, gardening, orienteering, hunting or forestry) would explain this pattern.

We hypothesise that the 2020 increase in TBE case was primarily due to human behavioural changes that resulted from the non-pharmaceutical interventions applied in response to the COVID-19 pandemic. Because of such interventions, which included movement restrictions and physical distancing, people may have engaged more in local outdoor activities, including gardening and walks in the countryside or forest. Data from Germany seem to support this hypothesis [14]. Nevertheless, other factors such as environmental drivers, including temperature [39] or outbreaks, may have contributed as well. For instance, in Germany, a higher tick abundance was noticed, which could have contributed to a higher exposure to tick bites. However, no European-wide increase in tick abundance was observed [40]. In 2020, in France, there was an alimentary TBE outbreak with 43 cases, which has partially contributed to the increase and illustrates that TBEV transmission is not solely related to tick bites [41].

Despite the comprehensive nature of the surveillance systems in place in EU/EEA countries, the TBE cases described in this study only represent a small fraction of the actual number of infections occurring in the EU. First, the EU case definition requires countries to report only laboratory diagnosed cases with clinical manifestations. A large part of the TBEV infections remain asymptomatic, hence are not detected, and individuals with mild forms of the disease may not be appropriately diagnosed. Second, not all EU/EEA countries were included in our study and some cases had to be excluded as epidemiological information was incomplete. Third, despite the existence of an EU case definition, France and Germany have been following a slightly different case definition [6]. This emphasises that comparison between countries should be undertaken with caution due to inherent differences in surveillance systems and testing practices. Despite these limitations, we consider our conclusions to be valid as the countries included were sufficiently representative to draw conclusions for the EU/EEA and these limitations were constant over time.

Finally, we have provided the highest level of geographical detail available (NUTS-3 regions) but acknowledge that the risk is not equally spread throughout these regions. In fact, the distribution of TBEV is generally very focal and patchy, as illustrated in a recent study in Southern Germany [42].

### Conclusion

We provided a detailed update on the epidemiology of TBE in the EU/EEA from 2012 to 2020. During this period, we observed a statistically significant increase in TBE cases, as well as a north-west bound spread on continental Europe. As the TBE epidemiological situation in the EU/EEA is changing, close monitoring of such changes is required to timely adapt vaccination recommendations. Further analyses to identify populations and geographical areas where vaccination programmes can be of benefit are needed.

## *Note

The United Kingdom (UK) left the European Union on 31 January 2020 and therefore was excluded from the study.

## Supplementary Data

1407000 Supplementary Material
