# Supplementary material for: Spatiotemporal spread of tick-borne encephalitis in the EU/EEA, 2012 to 2020
Source: Euro Surveill. 2023 Mar 16;28(11):2200543. doi: 10.2807/1560-7917.ES.2023.28.11.2200543 (PMC10021474; doi:10.2807/1560-7917.ES.2023.28.11.2200543)
Supplement: Supplementary Material [file 22-00543_GOSSNER_Supplement.pdf]

Supplementary material

Spatiotemporal spread of tick-borne encephalitis in the EU/EEA, 2012 to 2020

This supplementary material is hosted by *Eurosurveillance* as supporting information alongside the article 'Spatiotemporal spread of tick-borne encephalitis in the EU/EEA, 2012 to 2020', on behalf of the authors, who remain responsible for the accuracy and appropriateness of the content. The same standards for ethics, copyright, attributions and permissions as for the article apply. Supplements are not edited by *Eurosurveillance* and the journal is not responsible for the maintenance of any links or email addresses provided therein.

Supplementary material S1. Study flowchart

EU/EEA member states have reported 26,043 TBE cases with onset between 1 January 2012 and 31 December 2020. After data cleaning, 855 cases had an unknown importation status and were excluded. As described in the methods, 318 cases reported by Finland with missing importation status were classified as autochthonous in agreement with the Finnish Institute for Health and Welfare. A total of 214 cases were additionally excluded since they were reported by countries that did not report case-based data every year. These countries are Belgium, Bulgaria, Croatia, Cyprus, Denmark, Italy, Luxembourg, Malta, Portugal, the United Kingdom, Iceland, and Liechtenstein.

Supplementary Figure S1. Study flowchart representing inclusion and exclusion of cases

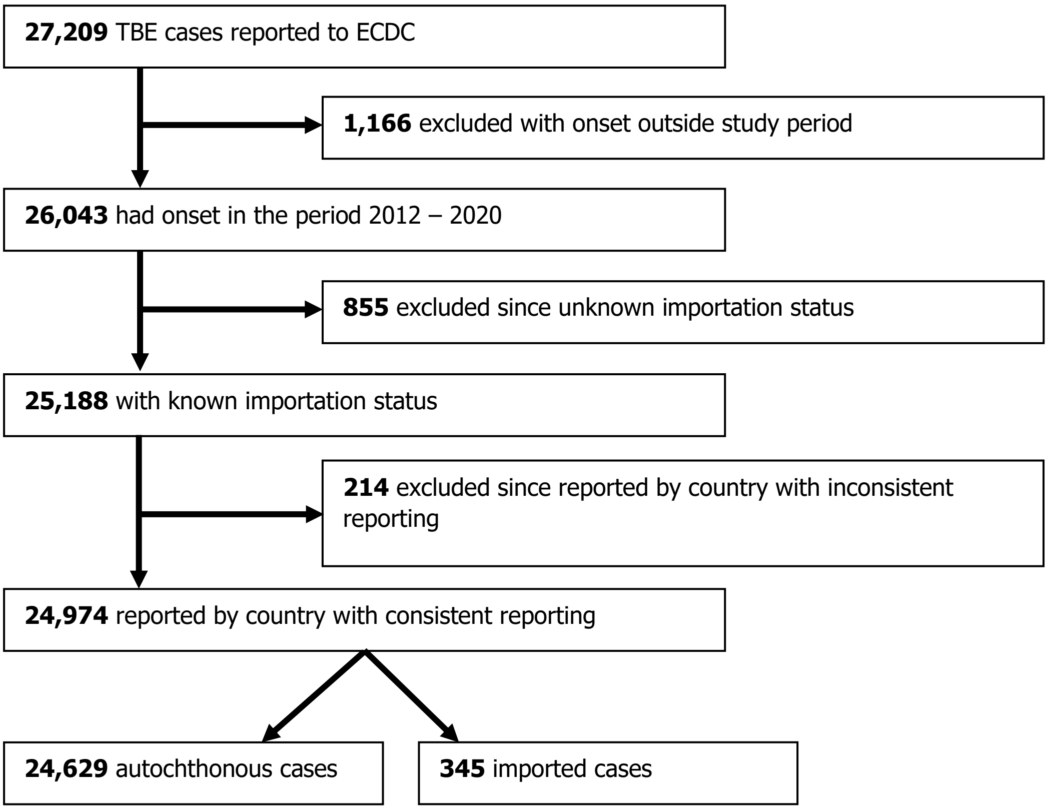

Supplementary material S2. Imputation rules spatial analysis

Whenever the place of infection was not available for autochthonous cases, the place of residence and the place of notification were taken as proxies (in respective order, depending on availability). To support this imputation, the different location variables were compared where possible. Comparison between the place of infection and place of residence shows a concordance of 90.21% at NUTS-3 level (n = 16,999) and 94.56% at NUTS-2 level (n = 18,002). Comparison between the place of infection and the place of notification shows a concordance of 90.48% at NUTS-3 level (n = 15,781) and 94.45% at NUTS-2 level (n = 18,004). Table 1 and 2 indicate that the concordance levels are similar in the different countries, except for Latvia. However, it should be noted that very few cases could be tested in this country.

Supplementary Table S1. Concordance place of infection and place of residence (NUTS-3), by country

Country	Concordance percentage	Concordance count	Total compared
**Czechia**	92.8	5,114	5,513
**Germany**	88.6	2,865	3,232
**Estonia**	84	441	525
**Greece**	100	2	2
**Finland**	84	216	257
**Hungary**	96.4	163	169
**Lithuania**	91.5	3,432	3,751
**Latvia**	52.2	24	46
**Poland**	87.3	1,065	1,220
**Romania**	100	9	9
**Sweden**	87.4	1,867	2,135
**Slovenia**	89.7	35	39
**Slovakia**	100	101	101
**Total**	90.2	15,334	16,999

Supplementary Table S2. Concordance place of infection and place of notification (NUTS-3), by country

Country	Concordance percentage	Concordance count	Total compared
**Czechia**	92.7	5,111	5,513
**Estonia**	84	441	525
**Germany**	88.6	2,865	3,232
**Greece**	50	1	2
**Hungary**	96.4	163	169
**Latvia**	52.2	24	46
**Lithuania**	91.5	3,434	3,751
**Romania**	100	9	9
**Slovakia**	100	101	101
**Slovenia**	89.7	35	39
**Sweden**	87.5	2,095	2,394
**Total**	90.5	14,279	15,781

The extent to which TBE cases from different countries have been affected by the imputation is presented in Table 3. In addition, the table illustrates the number of cases without geographical information at the NUTS-2 or NUTS-3 level.

Supplementary Table S3. Imputation rules per country

Country	Level	% Original	% Proxy	% Unknown	Count unknown
**Austria**	NUTS-2	100	0	0	0
**Czechia**	NUTS-3	99.8	0.2	0	0
**Estonia**	NUTS-3	58.9	41.1	0	0
**Finland**	NUTS-3	44.5	54.8	0.7	4
**France**	NUTS-2	66.7	0	33.3	33
**Germany**	NUTS-3	97.7	2.0	0.3	10
**Greece**	NUTS-3	100	0	0	0
**Hungary**	NUTS-3	67.3	32.3	0.4	1
**Latvia**	NUTS-3	2.7	97.3	0	0
**Lithuania**	NUTS-3	89.4	10.6	0	0
**Netherlands**	Country	0	0	0	0
**Norway**	Country	0	0	0	0
**Poland**	NUTS-3	63.1	36.7	0.2	3
**Romania**	NUTS-3	100	0	0	0
**Slovakia**	NUTS-3	8.4	91.6	0	0
**Slovenia**	NUTS-3	3.1	96.9	0	0
**Sweden**	NUTS-3	94.9	5.0	0.1	2

Legend: Level (detail of geographical presentation); % Original (percentage of cases with originally reported place of infection); % Proxy (percentage of cases with proxy variable used); % Unknown (percentage of cases without information about location after imputation); Count unknown (number of cases without information about location after imputation)

Supplementary material S3. Verification of tick-borne encephalitis virus transmission in scientific literature

Place of infection was the primary variable of interest for all cases in spatial analyses. Whenever this variable was not available for autochthonous cases, place of residence and place of notification were taken as proxies (in respective order, depending on availability). To minimize bias, only NUTS regions with verified TBE virus transmission were eligible as a proxy. Even though the following NUTS levels were not originally reported as place of infection in our database (i.e., no documented TBE transmission in the TESSy database), they were accepted as proxies based on the cited scientific evidence indicating TBE virus transmission:

- Latvia: Rīga (LV006), Vidzeme (LV008), Zemgale (LV009) [1]
- Poland: Nowosądecki (PL218), Bielski (PL225), Gorzowski (PL431), Trójmiejski (PL633) [2,3]
- Slovenia: Savinjska (SI034), Zasavska (SI035), Posavska (SI036), Obalno-kraška (SI044) [4].

References:

1. Zavadska D, Odzelevica Z, Karelis G, Liepina L, Litauniece ZA, Bormane A, et al. Tick-borne encephalitis: A 43-year summary of epidemiological and clinical data from Latvia (1973 to 2016). PLoS One. 2018;13(11):e0204844.
2. Stefanoff P, Zielicka-Hardy A, Hlebowicz M, Konior R, Lipowski D, Szenborn L, et al. New endemic foci of tick-borne encephalitis (TBE) identified in districts where testing for TBE was not available before 2009 in Poland. Parasit Vectors. 2013 Jun 18;6:180.
3. Stefanoff P, Rubikowska B, Bratkowski J, Ustrnul Z, Vanwambeke SO, Rosinska M. A Predictive Model Has Identified Tick-Borne Encephalitis High-Risk Areas in Regions Where No Cases Were Reported Previously, Poland, 1999-2012. Int J Environ Res Public Health.
4. Grgic-Vitek M, Klavs I. High burden of tick-borne encephalitis in Slovenia--challenge for vaccination policy. Vaccine. 2011 Jul 18;29(32):5178-83.

Supplementary material S4. Regional spatial analysis

We assessed 24,974 TBE cases which were reported by nineteen EU/EEA countries between 2012 and 2020. Out of those, 24,629 were autochthonous cases for which a geographical analysis at regional level was performed. The following countries reported geographical information at NUTS-3 level: Czechia, Germany, Estonia, Greece, Spain, Finland, Hungary, Ireland, Lithuania, Latvia, Poland, Romania, Sweden, Slovenia, and Slovakia. Austria and France reported geographical information at NUTS-2 level. The Netherlands and Norway only reported cases at country level and are therefore not included in the regional analysis. As a result, 786 different NUTS levels were evaluated in the geographical analysis of autochthonous cases.

A total of 53 autochthonous cases did not have information at regional level and are therefore not included in the regional analysis. Table 1 provides an overview of how these cases are distributed across the reporting countries and the study years.

Supplementary Table S4. Number of cases with missing geographical information at regional level

Country	2012	2013	2014	2015	2016	2017	2018	2019	2020	Total
**Finland**	0	2	1	0	1	0	0	0	0	4
**France**	0	1	7	7	18	0	0	0	0	33
**Germany**	0	2	1	0	0	2	4	1	0	10
**Hungary**	0	1	0	0	0	0	0	0	0	1
**Poland**	0	1	0	0	0	0	2	0	0	3
**Sweden**	0	0	0	0	0	1	1	0	0	2

Supplementary material S5. Notification rate per year and region

The table below presents the 786 different NUTS-2 and NUTS-3 levels included in the geographical analysis. The Netherlands and Norway are not included. The notification rate per year and in total (NR) is shown, as well as the total number of cases.

Supplementary Table S5. Notification rate per year and region

Code	Name	2012	2013	2014	2015	2016	2017	2018	2019	2020	Cases	NR
**AT**	**AUSTRIA**											
AT33	Tirol	1.7	3.4	2.6	2.1	3	4.4	4.1	2.8	7.8	236	3.6
AT31	Oberösterreich	0.8	2.5	1.7	1.1	1.9	2.9	4.6	2.6	3.8	320	2.5
AT22	Steiermark	1	1.2	0.9	1.8	1.1	1.2	1.9	1	3.4	168	1.5
AT32	Salzburg	0.6	0.9	0.6	0.6	1.3	1.3	2	1.6	4.5	73	1.5
AT21	Kärnten	0.9	1.4	2	0.4	0.7	0.7	1.6	0.7	2.9	63	1.2
AT34	Vorarlberg	0.5	0.5	0.5	0.8	0.5	1	1	1	2.8	34	1
AT12	Niederösterreich	0.1	0.4	0.2	0.3	0.4	0.7	1	0.5	1.4	84	0.6
AT13	Wien	0.1	0.1	0.1	0	0.2	0.2	0.2	0.2	0.2	21	0.1
AT11	Burgenland	0	0.3	0.3	0	0.3	0	0	0.3	0	4	0.1
**CZ**	**CZECHIA**											
CZ031	Jihočeský kraj	21.4	21.7	11.3	10.7	18.2	20.4	19.4	17	22	1035	18
CZ063	Kraj Vysočina	11.3	10.6	9.4	9.8	13.2	15.3	16.5	18.5	23.3	652	14.2
CZ032	Plzeňský kraj	11	9.8	6.4	4.5	8.8	7.3	9.6	5.8	9.5	421	8.1
CZ053	Pardubický kraj	5.2	4.8	3.9	4.4	5.8	9.5	7.5	11.7	13.8	346	7.4
CZ071	Olomoucký kraj	4.2	7.8	5.5	2.5	4.2	7.6	11.8	13	7	404	7.1
CZ041	Karlovarský kraj	3.6	4.6	4	3.7	5.4	9.8	4.7	8.1	9.2	158	5.9
CZ072	Zlínský kraj	3.7	4.2	2.7	3.1	4.8	4.1	7.4	8.8	13.6	306	5.8

Code	Name	2012	2013	2014	2015	2016	2017	2018	2019	2020	Cases	NR
CZ042	Ústecký kraj	5.6	5.4	4.5	3.6	4.6	6.8	5.5	4.6	3.9	367	5
CZ020	Středočeský kraj	5.7	5.7	3.5	3	4.2	5.1	4.9	5.1	3.5	540	4.5
CZ051	Liberecký kraj	3	4.1	2.5	1.6	5.5	5.4	4.5	5.9	7	174	4.4
CZ064	Jihomoravský kraj	2.8	3.9	1.9	2	3.1	2.9	4.2	4.6	7	382	3.6
CZ080	Moravskoslezský kraj	2.5	2.9	2.7	1.5	2.8	4	4	6.2	4.4	375	3.4
CZ052	Královéhradecký kraj	1.8	1.8	1.1	1.1	3.3	3.3	3.6	3.8	6.5	145	2.9
CZ010	Hlavní město Praha	1.7	2.5	1.2	0.7	1.3	3	2.4	2.8	1.4	217	1.9
**EE**	**ESTONIA**											
EE004	Lääne-Eesti	45.5	31.6	23	24.4	20.6	32.1	33.5	22.8	18.8	377	28
EE008	Lõuna-Eesti	16.5	10.5	4	10	6	2.5	4.1	5.4	7.9	215	7.5
EE009	Kesk-Eesti	6.3	4.7	8	8	0.8	0.8	4.8	4.9	6.5	56	5
EE001	Põhja-Eesti	5.7	4.2	3.9	5.1	4.2	4.1	1.5	3.8	1.3	195	3.7
EE00A	Kirde-Eesti	10.4	2	2.7	4	4.1	3.5	3.6	2.2	0	48	3.7
**FI**	**FINLAND**											
FI200	Åland	21.2	24.6	10.5	31.1	13.8	37.6	30.5	26.9	40.2	69	26.4
FI1C5	Etelä-Karjala	1.5	2.3	6	6.1	3.8	3.8	3.1	3.9	1.6	42	3.6
FI1C1	Varsinais-Suomi	1.5	0.6	2.1	3	3.4	6.3	4.4	3.3	2.7	130	3
FI1D7	Lappi	3.3	1.1	1.6	1.6	1.7	5	0	0.6	1.7	30	1.8
FI1B1	Helsinki-Uusimaa	0.7	0.7	0.9	1.7	1.5	1.2	2.1	1.6	2.5	209	1.4
FI1C4	Kymenlaakso	1.1	0	1.7	1.1	0.6	1.1	0	1.1	1.8	15	0.9
FI1D3	Pohjois-Karjala	0.6	0.6	0.6	0.6	0.6	0	0	1.9	2.5	12	0.8
FI1D2	Pohjois-Savo	0	0	0.8	0.8	0	0.8	1.6	0.4	0.8	13	0.6
FI1D9	Pohjois-Pohjanmaa	0.2	1	0.2	0	0.5	1	0	0.7	0.7	18	0.5

Code	Name	2012	2013	2014	2015	2016	2017	2018	2019	2020	Cases	NR
FI195	Pohjanmaa	0.6	0.6	0	0	0.6	0.6	1.1	0	0.6	7	0.4
FI1D1	Etelä-Savo	0.7	0	0	0	0	0	1.4	0	1.4	5	0.4
FI1D5	Keski-Pohjanmaa	1.5	0	0	0	0	0	0	1.5	0	2	0.3
FI196	Satakunta	0.4	0.4	0.4	0.4	0.4	0.4	0	0	0	6	0.3
FI197	Pirkanmaa	0	0.6	0	0	0	0	0.2	0.6	0.6	10	0.2
FI1C2	Kanta-Häme	0	0	0	0	1.1	0	0.6	0	0	3	0.2
FI1C3	Päijät-Häme	0	0	0	0.5	0	0	0	0	0.5	2	0.1
FI193	Keski-Suomi	0	0	0	0	0	0	0	0	0	0	0
FI194	Etelä-Pohjanmaa	0	0	0	0	0	0	0	0	0	0	0
FI1D8	Kainuu	0	0	0	0	0	0	0	0	0	0	0
**FR**	**FRANCE**											
FRF1	Alsace	0	0	0	0	0	0	0.8	0	0	16	0.1
FRK2	Rhône-Alpes	0	0	0	0	0	0	0	0	0.6	45	0.1
FRK1	Auvergne	0	0	0	0	0	0.1	0.1	0	0	2	0
FRF3	Lorraine	0	0	0	0	0	0	0	0	0	1	0
FRC2	Franche-Comté	0	0	0	0	0	0	0	0.1	0	1	0
FRF2	Champagne-Ardenne	0	0	0	0	0	0	0	0.1	0	1	0
FR10	Ile-de-France	0	0	0	0	0	0	0	0	0	0	0
FRB0	Centre — Val de Loire	0	0	0	0	0	0	0	0	0	0	0
FRC1	Bourgogne	0	0	0	0	0	0	0	0	0	0	0
FRD1	Basse-Normandie	0	0	0	0	0	0	0	0	0	0	0
FRD2	Haute-Normandie	0	0	0	0	0	0	0	0	0	0	0
FRE1	Nord-Pas de Calais	0	0	0	0	0	0	0	0	0	0	0

Code	Name	2012	2013	2014	2015	2016	2017	2018	2019	2020	Cases	NR
FRE2	Picardie	0	0	0	0	0	0	0	0	0	0	0
FRG0	Pays de la Loire	0	0	0	0	0	0	0	0	0	0	0
FRH0	Bretagne	0	0	0	0	0	0	0	0	0	0	0
FRI1	Aquitaine	0	0	0	0	0	0	0	0	0	0	0
FRI2	Limousin	0	0	0	0	0	0	0	0	0	0	0
FRI3	Poitou-Charentes	0	0	0	0	0	0	0	0	0	0	0
FRJ1	Languedoc-Roussillon	0	0	0	0	0	0	0	0	0	0	0
FRJ2	Midi-Pyrénées	0	0	0	0	0	0	0	0	0	0	0
FRL0	Provence-Alpes-Côte d’Azur	0	0	0	0	0	0	0	0	0	0	0
FRM0	Corse	0	0	0	0	0	0	0	0	0	0	0
FRY1	Guadeloupe	0	0	0	0	0	0	0	0	0	0	0
FRY2	Martinique	0	0	0	0	0	0	0	0	0	0	0
FRY3	Guyane	0	0	0	0	0	0	0	0	0	0	0
FRY4	La Réunion	0	0	0	0	0	0	0	0	0	0	0
FRY5	Mayotte	0	0	0	0	0	0	0	0	0	0	0
**DE**	**GERMANY**											
DE234	Amberg-Sulzbach	3.9	13.6	7.8	8.7	8.7	10.7	13.6	10.7	9.7	90	9.7
DE12A	Calw	3.3	7.3	4	2	10.9	6.4	11.4	5.7	11.9	98	7
DE239	Schwandorf	3.5	4.2	6.3	4.2	6.9	6.2	11.6	6.1	9.5	85	6.5
DE225	Freyung-Grafenau	1.3	3.9	3.9	7.7	5.1	12.8	8.9	1.3	12.8	45	6.4
DE258	Fürth, Landkreis	0	11.4	3.5	2.6	9.6	6.9	6.9	7.7	4.2	61	5.9
DE12C	Freudenstadt	1.7	5.2	1.7	1.7	3.4	5.1	8.5	5.1	18.6	60	5.7
DE237	Neustadt a. d. Waldnaab	6.2	6.3	2.1	4.2	2.1	5.3	11.6	4.2	7.4	47	5.5

Code	Name	2012	2013	2014	2015	2016	2017	2018	2019	2020	Cases	NR
DE135	Rottweil	1.5	6.6	0.7	2.2	4.4	6.5	5	2.1	15	61	4.9
DE215	Berchtesgadener Land	2	3.9	4.9	5.8	3.9	5.7	6.7	4.7	1.9	41	4.4
DE143	Zollernalbkreis	2.7	3.8	1.1	0	2.1	4.8	6.4	3.7	11.1	67	4
DE148	Ravensburg	0.7	2.6	1.1	2.9	2.1	6.8	8.1	4.6	6.7	100	4
DE149	Sigmaringen	0.8	1.6	3.9	3.9	1.5	0	5.4	4.6	14.5	47	4
DE21M	Traunstein	0.6	3	1.2	4.1	2.9	5.1	6.8	2.8	7.9	60	3.8
DE235	Cham	1.6	4.8	0.8	0.8	1.6	9.4	5.5	3.1	6.2	43	3.8
DE134	Ortenaukreis	2.2	5.1	1.7	1	3.8	4.2	7.5	1.9	6	141	3.7
DE246	Bayreuth, Landkreis	3.8	4.8	0	1	2.9	4.8	4.8	4.8	6.8	35	3.7
DE222	Passau, Kreisfreie Stadt	0	6.1	8.1	10	0	2	1.9	0	5.7	17	3.7
DE231	Amberg, Kreisfreie Stadt	0	4.8	4.8	4.8	0	9.4	2.4	2.4	4.7	14	3.7
DE24A	Kronach	1.4	8.7	1.5	4.4	2.9	0	1.5	1.5	9	21	3.4
DE147	Bodenseekreis	2	4.4	1.4	2.4	2.8	2.4	6.1	4.2	3.7	62	3.3
DE229	Regen	1.3	3.9	0	0	2.6	2.6	6.4	2.6	9	22	3.2
DE228	Passau, Landkreis	0.5	2.7	6.5	2.7	2.1	6.9	2.6	0.5	4.7	55	3.2
DE23A	Tirschenreuth	1.3	0	2.7	0	2.7	4.1	1.4	11	5.6	21	3.2
DE71B	Odenwaldkreis	2.1	2.1	3.1	3.1	6.2	2.1	3.1	4.1	2.1	27	3.1
DEG04	Suhl, Kreisfreie Stadt	0	0	0	0	5.4	0	0	14.3	8.2	10	3.1
DE259	Nürnberger Land	1.8	3.6	1.2	0	2.4	3	3.5	3.5	8.2	46	3
DE13A	Waldshut	0	3.7	3.6	0	1.2	3.5	2.9	2.3	8.2	43	2.9
DE22A	Rottal-Inn	1.7	2.5	3.4	5.9	0	7.5	1.7	1.7	0.8	30	2.8
DE27A	Lindau (Bodensee)	0	1.3	2.5	0	0	3.7	4.9	6.1	6.1	20	2.8
DE256	Ansbach, Landkreis	0.6	3.9	1.7	3.3	3.9	3.3	1.1	3.3	1.1	40	2.5

Code	Name	2012	2013	2014	2015	2016	2017	2018	2019	2020	Cases	NR
DE238	Regensburg, Landkreis	1.1	1.6	2.7	3.2	2.6	3.1	1.6	1	4.1	40	2.4
DE248	Forchheim	3.5	1.8	2.6	0	0	6.1	1.7	3.5	1.7	24	2.3
DE24B	Kulmbach	4.1	0	4.1	0	2.8	1.4	4.2	1.4	2.8	15	2.3
DE27C	Unterallgäu	0	0	0.7	0	2.9	3.5	1.4	4.9	6.9	29	2.3
DE25B	Roth	4.1	4.1	3.2	2.4	0	0	3.2	0	3.2	25	2.2
DE133	Emmendingen	1.3	3.2	2.5	0.6	2.5	0.6	4.2	1.8	2.4	31	2.1
DE249	Hof, Landkreis	1	2	1	0	0	5.2	2.1	2.1	4.2	17	2
DE129	Pforzheim, Stadtkreis	0.9	3.4	3.4	0.8	1.6	0.8	3.2	2.4	1.6	22	2
DE26A	Main-Spessart	3.9	5.5	2.4	2.4	2.4	0	0	0	1.6	23	2
DED44	Vogtlandkreis	0.4	1.7	3.9	0.9	0.4	1.3	1.3	3.5	4.4	41	2
DE255	Schwabach, Kreisfreie Stadt	0	5.1	0	0	2.5	0	4.9	4.9	0	7	1.9
DE21K	Rosenheim, Landkreis	0	2	0	0	3.1	4.3	3.1	2.7	1.9	44	1.9
DE245	Bamberg, Landkreis	1.4	0.7	0.7	1.4	4.8	4.1	0	2	1.4	24	1.8
DE138	Konstanz	1.9	3	0.7	0.7	1.8	1.1	3.5	1.8	1.8	45	1.8
DE142	Tübingen, Landkreis	0.5	3.7	0.9	0.5	1.4	3.6	2.2	1.3	2.2	36	1.8
DE27E	Oberallgäu	0.7	0.7	2.7	1.3	1.3	0.7	2.6	1.3	5.1	25	1.8
DE236	Neumarkt i. d. OPf.	0	1.6	0	0.8	3.1	2.3	3.8	1.5	2.2	20	1.7
DE137	Tuttlingen	0	1.5	0.8	0	1.5	3.6	0.7	1.4	5.7	21	1.7
DE136	Schwarzwald-Baar-Kreis	0	0	0.5	0	1.4	2.4	3.8	0.5	6.6	32	1.7
DE224	Deggendorf	0	0	0.9	0.9	0	2.5	5	1.7	4.2	18	1.7
DE715	Bergstraße	1.1	2.7	0.4	0.8	3	0.4	3	0.7	1.9	37	1.6
DE22B	Straubing-Bogen	0	2.1	1	0	2	6	1	1	1	14	1.6
DE141	Reutlingen	0	2.2	0.4	0	1.1	1.4	1.4	2.4	4.9	39	1.5

Code	Name	2012	2013	2014	2015	2016	2017	2018	2019	2020	Cases	NR
DE242	Bayreuth, Kreisfreie Stadt	0	0	1.4	1.4	1.4	2.7	2.7	1.3	2.7	10	1.5
DE11D	Ostalbkreis	0.7	1.3	0.7	0.7	1.9	1.6	1.9	2.5	1.6	40	1.4
DE132	Breisgau-Hochschwarzwald	2	0.4	0.8	0	1.9	1.1	3	0.4	2.3	31	1.4
DE25C	Weißenburg-Gunzenhausen	1.1	3.2	2.2	0	4.3	0	0	2.1	0	12	1.4
DE253	Fürth, Kreisfreie Stadt	0	1.7	2.5	0.8	2.4	4	0.8	0	0.8	16	1.4
DE121	Baden-Baden, Stadtkreis	0	0	0	0	1.9	1.8	3.7	1.8	3.6	7	1.4
DE254	Nürnberg, Kreisfreie Stadt	0.8	1	1	1.2	1.4	1.6	1	2.1	1.9	61	1.3
DE131	Freiburg im Breisgau, Stadtkreis	0.9	0.9	0.9	0.9	0.4	0.9	2.2	1.3	3.5	27	1.3
DE221	Landshut, Kreisfreie Stadt	0	3.1	0	1.5	0	0	1.4	1.4	4.1	8	1.3
DE216	Bad Tölz-Wolfratshausen	0.8	0	0	0	0	2.4	0.8	1.6	4.7	13	1.2
DE12B	Enzkreis	0	3.1	0.5	0	0.5	2	1.5	1	2	21	1.2
DE112	Böblingen	0.8	2.2	0.3	0.5	0	1.6	3.3	0	1.3	38	1.1
DE21G	Mühldorf a. Inn	0.9	0.9	0.9	0.9	0.9	1.8	0.9	1.7	0.9	11	1.1
DEG03	Jena, Kreisfreie Stadt	0	3.7	0	0	0.9	0.9	2.7	1.8	0	11	1.1
DE214	Altötting	0.9	0.9	0	1.9	0	0.9	3.6	0.9	0	10	1
DE252	Erlangen, Kreisfreie Stadt	1	0.9	0	0.9	0.9	1.8	0	0.9	2.7	10	1
DE227	Landshut, Landkreis	0.7	2	0	1.3	0	0	1.9	1.3	1.9	14	1
DE25A	Neustadt a. d. Aisch-Bad Windsheim	0	1	0	1	2	3	0	0	2	9	1
DE233	Weiden i. d. Opf, Kreisfreie Stadt	0	0	0	2.4	2.4	2.4	2.4	0	0	4	1
DE274	Memmingen, Kreisfreie Stadt	0	0	0	0	0	0	4.6	0	4.5	4	1
DE269	Miltenberg	2.3	0	0	0.8	0.8	1.6	1.6	0.8	0.8	11	0.9
DE21A	Erding	0.8	0.8	0	1.5	0.8	1.5	0.7	0.7	1.4	11	0.9
DE146	Biberach	0	1.1	0	0	0	1	2.5	1.5	2	16	0.9

Code	Name	2012	2013	2014	2015	2016	2017	2018	2019	2020	Cases	NR
DE21F	Miesbach	0	0	1	0	0	2	2	2	1	8	0.9
DE264	Aschaffenburg, Landkreis	2.3	1.2	0	1.2	0.6	1.1	0.6	0	0	12	0.8
DE21L	Starnberg	1.6	0	0	0	1.5	2.2	0.7	0.7	0.7	10	0.8
DE716	Darmstadt-Dieburg	0.3	0.3	0.3	0	0.3	0	2	2.4	1.3	21	0.8
DE241	Bamberg, Kreisfreie Stadt	0	2.8	0	1.4	1.4	0	0	0	1.3	5	0.8
DE257	Erlangen-Höchstadt	0	2.3	0	1.5	0.8	0.7	0	0.7	1.5	10	0.8
DE139	Lörrach	0	0.9	0.4	0	0.9	2.6	0.9	0.4	0.9	16	0.8
DEG0L	Greiz	0	0	0	0	0	2	1	1	3.1	7	0.8
DEB15	Birkenfeld	3.6	0	0	1.2	0	0	1.2	0	0	5	0.7
DE22C	Dingolfing-Landau	1.1	0	1.1	1.1	2.1	0	1	0	0	6	0.7
DE111	Stuttgart, Stadtkreis	0.5	2	0.3	0.2	0	0.5	0.9	0.3	1.9	41	0.7
DE123	Karlsruhe, Landkreis	0.7	0	0.7	0.2	0.5	0.4	1.1	1.4	0.9	26	0.7
DE226	Kelheim	0.9	0	0.9	0.9	0.8	1.7	0	0	0.8	7	0.7
DE265	Bad Kissingen	0	1	0	0	0	1.9	1	1.9	0	6	0.7
DE27B	Ostallgäu	0	1.5	0	0.7	0.7	0	2.1	0	0.7	8	0.7
DEG0F	Ilm-Kreis	0	0.9	0.9	0	1.8	0	0	0.9	1.9	7	0.7
DE21J	Pfaffenhofen a. d. Ilm	0	0	0.8	0.8	0	0.8	3.2	0.8	0	8	0.7
DED2F	Sächsische Schweiz-Osterzgebirge	0	0	0	0.4	0.8	0.8	0.4	0.8	2.9	15	0.7
DE24C	Lichtenfels	0	0	0	0	3	1.5	0	1.5	0	4	0.7
DE128	Rhein-Neckar-Kreis	0.9	1.7	0.4	0.2	0.4	0.6	0.2	0.7	0	27	0.6
DE116	Rems-Murr-Kreis	0.5	0	0.5	0.5	0	0.5	2.1	0.5	0.9	23	0.6
DE113	Esslingen	0	1.6	0.2	0	0.4	0.6	1.5	0.9	0.2	28	0.6
DE219	Eichstätt	0	0.8	0	0	0	2.3	1.5	0	0.8	7	0.6

Code	Name	2012	2013	2014	2015	2016	2017	2018	2019	2020	Cases	NR
DE124	Rastatt	0	0.9	0	0	0	0.4	0.9	1.3	1.7	12	0.6
DE21B	Freising	0	1.2	1.2	0	0	0	1.1	0	1.7	9	0.6
DE144	Ulm, Stadtkreis	0	0.8	0	0	0	0.8	1.6	0	1.6	6	0.6
DE21N	Weilheim-Schongau	0	0	0	0.8	0	0.8	0.7	0	3	7	0.6
DEG0H	Sonneberg	0	0	0	0	0	0	3.5	0	1.7	3	0.6
DE724	Marburg-Biedenkopf	0.4	0	0.8	0.4	0	0.4	1.2	0.4	0.4	10	0.5
DE267	Haßberge	1.2	1.2	0	1.2	0	1.2	0	0	0	4	0.5
DE71C	Offenbach, Landkreis	0.6	0.6	1.2	0.3	0	1.4	0.3	0.3	0	16	0.5
DE125	Heidelberg, Stadtkreis	0.7	0.7	1.3	0.7	0	0.6	0.6	0	0	7	0.5
DE244	Hof, Kreisfreie Stadt	0	2.2	0	0	0	0	0	2.2	0	2	0.5
DE145	Alb-Donau-Kreis	0	0	1.1	0	0	1	1	0	1	8	0.5
DE279	Neu-Ulm	0	0	0	0.6	0	2.3	0.6	0	0.6	7	0.5
DE949	Emsland	0	0	0	0	0.6	0.6	1.2	1.8	0.3	15	0.5
DEG0K	Saale-Orla-Kreis	0	0	0	0	0	1.2	1.2	0	2.5	4	0.5
DEG02	Gera, Kreisfreie Stadt	0	0	0	0	0	1.1	1	0	2.1	4	0.5
DE11C	Heidenheim	0.8	0	0.8	0	0	0.8	0.8	0	0.8	5	0.4
DE232	Regensburg, Kreisfreie Stadt	1.5	0	0	0.7	0	0	0.7	0	0.7	5	0.4
DED2C	Bautzen	0	0.6	0.7	0	0.7	0.3	0.3	1	0.3	12	0.4
DE24D	Wunsiedel i. Fichtelgebirge	0	1.3	0	1.4	0	0	0	0	1.4	3	0.4
DE119	Hohenlohekreis	0	0.9	0	0.9	0.9	0	0	0	0.9	4	0.4
DE247	Coburg, Landkreis	0	0	2.3	0	1.1	0	0	0	0	3	0.4
DE711	Darmstadt, Kreisfreie Stadt	0	0	1.3	0	0	1.3	0.6	0.6	0	6	0.4
DED42	Erzgebirgskreis	0	0	0.3	0	0	1.7	0	1.5	0.6	14	0.4

Code	Name	2012	2013	2014	2015	2016	2017	2018	2019	2020	Cases	NR
DEE01	Dessau-Roßlau, Kreisfreie Stadt	0	0	0	0	1.2	2.4	0	0	0	3	0.4
DE213	Rosenheim, Kreisfreie Stadt	0	0	0	0	1.6	1.6	0	0	0	2	0.4
DEG0I	Saalfeld-Rudolstadt	0	0	0	0	0.9	0.9	1.9	0	0	4	0.4
DEG0B	Schmalkalden-Meiningen	0	0	0	0	0.8	0.8	0.8	0	1.6	5	0.4
DEG0J	Saale-Holzland-Kreis	0	0	0	0	0	0	1.2	0	2.4	3	0.4
DE26B	Schweinfurt, Landkreis	0.9	0	0	0	0	0	0.9	0	0.9	3	0.3
DE122	Karlsruhe, Stadtkreis	0.7	0.7	0	0.3	0.7	0	0	0	0.3	8	0.3
DE11B	Main-Tauber-Kreis	1.5	0	0	0	0	0	0	0	1.5	4	0.3
DEB14	Bad Kreuznach	0.6	1.9	0	0	0	0	0.6	0	0	5	0.3
DED21	Dresden, Kreisfreie Stadt	0.2	0.2	0.2	0.2	0.2	0.6	0.4	0.4	0.7	16	0.3
DE266	Rhön-Grabfeld	0	1.2	0	0	0	0	0	0	1.3	2	0.3
DE732	Fulda	0	0.5	0	0	0.9	0.4	0.9	0	0.4	7	0.3
DE40G	Spree-Neiße	0	0.8	0.8	0	0	0	0.9	0	0	3	0.3
DE276	Augsburg, Landkreis	0	0.4	0.4	0	0	0.4	0	0	1.2	6	0.3
DE243	Coburg, Kreisfreie Stadt	0	0	2.4	0	0	0	0	0	0	1	0.3
DE21H	München, Landkreis	0	0	0	0.3	0.3	0	0.3	0.9	0.6	8	0.3
DE273	Kempten (Allgäu), Kreisfreie Stadt	0	0	0	1.5	0	0	0	0	1.4	2	0.3
DEB37	Pirmasens, Kreisfreie Stadt	0	0	0	0	2.5	0	0	0	0	1	0.3
DE218	Ebersberg	0	0	0	0	0	0	1.4	0	1.4	4	0.3
DEB3A	Zweibrücken, Kreisfreie Stadt	0	0	0	0	0	0	2.9	0	0	1	0.3
DE251	Ansbach, Kreisfreie Stadt	0	0	0	0	0	0	0	2.4	0	1	0.3
DE11A	Schwäbisch Hall	1.1	0.5	0	0	0	0	0	0.5	0	4	0.2
DE719	Main-Kinzig-Kreis	0.2	0.5	0.2	0	0	0.2	0.2	0	0	6	0.2

Code	Name	2012	2013	2014	2015	2016	2017	2018	2019	2020	Cases	NR
DE118	Heilbronn, Landkreis	0.3	0.3	0	0	0.6	0.3	0.3	0	0.3	7	0.2
DE27D	Donau-Ries	0.8	0	0	0	0.8	0	0	0	0	2	0.2
DE117	Heilbronn, Stadtkreis	0.9	0	0	0	0	0	0.8	0	0	2	0.2
DE217	Dachau	0.7	0	0	0.7	0	0	0	0	0.7	3	0.2
DE261	Aschaffenburg, Kreisfreie Stadt	1.5	0	0	0	0	0	0	0	0	1	0.2
DE271	Augsburg, Kreisfreie Stadt	0	1.5	0.7	0	0	0	0	0	0	6	0.2
DEB11	Koblenz, Kreisfreie Stadt	0	0.9	0	0	0	0	0.9	0	0	2	0.2
DE126	Mannheim, Stadtkreis	0	1.4	0	0	0	0	0	0.3	0	5	0.2
DE71D	Rheingau-Taunus-Kreis	0	1.1	0	0	0	0.5	0	0	0	3	0.2
DE263	Würzburg, Kreisfreie Stadt	0	0.8	0	0	0	0	0	0.8	0	2	0.2
DEA19	Solingen, Kreisfreie Stadt	0	0.6	0	0	0.6	0	0.6	0	0	3	0.2
DE40B	Oberspreewald-Lausitz	0	0.9	0	0	0	0	0.9	0	0	2	0.2
DEB13	Altenkirchen (Westerwald)	0	0	0.8	0	0	0	0	0	0.8	2	0.2
DE223	Straubing, Kreisfreie Stadt	0	0	2.2	0	0	0	0	0	0	1	0.2
DEG05	Weimar, Kreisfreie Stadt	0	0	1.6	0	0	0	0	0	0	1	0.2
DED2E	Meißen	0	0	0	0.4	0	0	0	0.4	0.8	4	0.2
DE114	Göppingen	0	0	0	0	0.4	0.4	0.4	0	0.8	5	0.2
DE402	Cottbus, Kreisfreie Stadt	0	0	0	0	1	0	0	1	0	2	0.2
DE927	Nienburg (Weser)	0	0	0	0	0.8	0.8	0	0	0	2	0.2
DEC03	Neunkirchen	0	0	0	0	0.8	0	0.8	0	0	2	0.2
DE40C	Oder-Spree	0	0	0	0	0.6	0	0	1.1	0	3	0.2
DEE05	Anhalt-Bitterfeld	0	0	0	0	0	0.6	0	1.2	0	3	0.2
DE21I	Neuburg-Schrobenhausen	0	0	0	0	0	0	1	0	1	2	0.2

Code	Name	2012	2013	2014	2015	2016	2017	2018	2019	2020	Cases	NR
DE278	Günzburg	0	0	0	0	0	0	0.8	0.8	0	2	0.2
DE277	Dillingen a.d. Donau	0	0	0	0	0	0	1	0	1	2	0.2
DE403	Frankfurt (Oder), Kreisfreie Stadt	0	0	0	0	0	0	0	0	1.7	1	0.2
DEG0E	Hildburghausen	0	0	0	0	0	0	0	0	1.6	1	0.2
DE929	Region Hannover	0.1	0	0	0.1	0.2	0	0	0.2	0.1	7	0.1
DE71E	Wetteraukreis	0.3	0	0	0	0	0.3	0	0	0	2	0.1
DE115	Ludwigsburg	0.2	0	0.2	0	0.2	0.4	0.4	0	0	7	0.1
DED41	Chemnitz, Kreisfreie Stadt	0.4	0	0	0	0	0	0	0.4	0	2	0.1
DE212	München, Kreisfreie Stadt	0.1	0	0	0.1	0.1	0.1	0.3	0.4	0.2	19	0.1
DE26C	Würzburg, Landkreis	1.3	0	0	0	0	0	0	0	0	2	0.1
DE735	Schwalm-Eder-Kreis	0	0.6	0	0	0	0	0	0	0	1	0.1
DEC05	Saarpfalz-Kreis	0	0.7	0	0	0	0	0.7	0	0	2	0.1
DE723	Limburg-Weilburg	0	0.6	0	0	0	0	0	0	0	1	0.1
DEA2C	Rhein-Sieg-Kreis	0	0.2	0	0	0	0.2	0	0	0.5	5	0.1
DEB1A	Rhein-Lahn-Kreis	0	0.8	0	0	0	0	0	0	0	1	0.1
DED2D	Görlitz	0	0.4	0.4	0	0	0	0.4	0	0	3	0.1
DEA37	Steinfurt	0	0.2	0.2	0	0	0	0.2	0	0	3	0.1
DED45	Zwickau	0	0.3	0	0	0	0	0	0.6	0	3	0.1
DE718	Hochtaunuskreis	0	0	0.4	0	0	0	0	0.4	0	2	0.1
DEG0G	Weimarer Land	0	0	1.2	0	0	0	0	0	0	1	0.1
DE93A	Uelzen	0	0	1.1	0	0	0	0	0	0	1	0.1
DE40I	Uckermark	0	0	0.8	0	0	0	0	0	0	1	0.1
DE717	Groß-Gerau	0	0	0.4	0.4	0	0.4	0	0	0	3	0.1

Code	Name	2012	2013	2014	2015	2016	2017	2018	2019	2020	Cases	NR
DE80L	Vorpommern-Rügen	0	0	0	0.4	0	0	0	0	0.4	2	0.1
DEB3C	Bad Dürkheim	0	0	0	0.8	0	0	0	0	0	1	0.1
DE931	Celle	0	0	0	0	0.6	0.6	0	0	0	2	0.1
DEB3F	Kaiserslautern, Landkreis	0	0	0	0	0.9	0	0	0	0	1	0.1
DED53	Nordsachsen	0	0	0	0	0.5	0.5	0	0	0	2	0.1
DEB32	Kaiserslautern, Kreisfreie Stadt	0	0	0	0	1	0	0	0	0	1	0.1
DEB12	Ahrweiler	0	0	0	0	0.8	0	0	0	0	1	0.1
DE913	Wolfsburg, Kreisfreie Stadt	0	0	0	0	0.8	0	0	0	0	1	0.1
DEE06	Jerichower Land	0	0	0	0	1.1	0	0	0	0	1	0.1
DE275	Aichach-Friedberg	0	0	0	0	0	0.8	0	0	0	1	0.1
DE737	Werra-Meißner-Kreis	0	0	0	0	0	1	0	0	0	1	0.1
DED43	Mittelsachsen	0	0	0	0	0	0.3	0	0.3	0.3	3	0.1
DE406	Dahme-Spreewald	0	0	0	0	0	0.6	0	0	0	1	0.1
DEA47	Paderborn	0	0	0	0	0	0.3	0.3	0	0.3	3	0.1
DE917	Helmstedt	0	0	0	0	0	0	1.1	0	0	1	0.1
DE21C	Fürstenfeldbruck	0	0	0	0	0	0	0.5	0	0.5	2	0.1
DEG06	Eichsfeld	0	0	0	0	0	0	1	0	0	1	0.1
DE21E	Landsberg am Lech	0	0	0	0	0	0	0.8	0	0	1	0.1
DE945	Wilhelmshaven, Kreisfreie Stadt	0	0	0	0	0	0	1.3	0	0	1	0.1
DE268	Kitzingen	0	0	0	0	0	0	0	1.1	0	1	0.1
DE932	Cuxhaven	0	0	0	0	0	0	0	0.5	0	1	0.1
DEE0B	Saalekreis	0	0	0	0	0	0	0	0.5	0	1	0.1
DE21D	Garmisch-Partenkirchen	0	0	0	0	0	0	0	0	1.1	1	0.1

Code	Name	2012	2013	2014	2015	2016	2017	2018	2019	2020	Cases	NR
DE409	Märkisch-Oderland	0	0	0	0	0	0	0	0	0.5	1	0.1
DEB23	Eifelkreis Bitburg-Prüm	0	0	0	0	0	0	0	0	1	1	0.1
DEA28	Euskirchen	0	0	0	0	0	0	0	0	0.5	1	0.1
DE80N	Vorpommern-Greifswald	0.4	0	0	0	0	0	0	0	0	1	0
DE300	Berlin	0	0	0	0	0	0	0	0	0	2	0
DE712	Frankfurt am Main, Kreisfreie Stadt	0	0.1	0	0	0	0	0	0	0	1	0
DE714	Wiesbaden, Kreisfreie Stadt	0	0.4	0	0	0	0	0	0	0	1	0
DEA34	Borken	0	0	0	0.3	0	0	0	0	0	1	0
DEA1F	Wesel	0	0	0	0.2	0	0	0	0	0	1	0
DEA27	Rhein-Erft-Kreis	0	0	0	0	0.2	0	0	0	0	1	0
DED52	Leipzig	0	0	0	0	0.4	0	0	0	0	1	0
DE722	Lahn-Dill-Kreis	0	0	0	0	0.4	0	0	0	0	1	0
DE80K	Landkreis Rostock	0	0	0	0	0.5	0	0	0	0	1	0
DEF09	Pinneberg	0	0	0	0	0	0.3	0	0	0	1	0
DE71A	Main-Taunus-Kreis	0	0	0	0	0	0.4	0	0	0	1	0
DE925	Hildesheim	0	0	0	0	0	0.4	0	0	0	1	0
DEA45	Lippe	0	0	0	0	0	0	0.3	0	0	1	0
DEA56	Ennepe-Ruhr-Kreis	0	0	0	0	0	0	0.3	0	0	1	0
DEA12	Duisburg, Kreisfreie Stadt	0	0	0	0	0	0	0.2	0	0	1	0
DEA33	Münster, Kreisfreie Stadt	0	0	0	0	0	0	0.3	0	0	1	0
DE91C	Göttingen	0	0	0	0	0	0	0	0.3	0	1	0
DEE02	Halle (Saale), Kreisfreie Stadt	0	0	0	0	0	0	0	0	0.4	1	0
DEE03	Magdeburg, Kreisfreie Stadt	0	0	0	0	0	0	0	0	0.4	1	0

Code	Name	2012	2013	2014	2015	2016	2017	2018	2019	2020	Cases	NR
DEA2A	Oberbergischer Kreis	0	0	0	0	0	0	0	0	0.4	1	0
DEC01	Regionalverband Saarbrücken	0	0	0	0	0	0	0	0	0.3	1	0
DE127	Neckar-Odenwald-Kreis	0	0	0	0	0	0	0	0	0	0	0
DE211	Ingolstadt, Kreisfreie Stadt	0	0	0	0	0	0	0	0	0	0	0
DE262	Schweinfurt, Kreisfreie Stadt	0	0	0	0	0	0	0	0	0	0	0
DE272	Kaufbeuren, Kreisfreie Stadt	0	0	0	0	0	0	0	0	0	0	0
DE401	Brandenburg an der Havel, Kreisfreie Stadt	0	0	0	0	0	0	0	0	0	0	0
DE404	Potsdam, Kreisfreie Stadt	0	0	0	0	0	0	0	0	0	0	0
DE405	Barnim	0	0	0	0	0	0	0	0	0	0	0
DE407	Elbe-Elster	0	0	0	0	0	0	0	0	0	0	0
DE408	Havelland	0	0	0	0	0	0	0	0	0	0	0
DE40A	Oberhavel	0	0	0	0	0	0	0	0	0	0	0
DE40D	Ostprignitz-Ruppin	0	0	0	0	0	0	0	0	0	0	0
DE40E	Potsdam-Mittelmark	0	0	0	0	0	0	0	0	0	0	0
DE40F	Prignitz	0	0	0	0	0	0	0	0	0	0	0
DE40H	Teltow-Fläming	0	0	0	0	0	0	0	0	0	0	0
DE501	Bremen, Kreisfreie Stadt	0	0	0	0	0	0	0	0	0	0	0
DE502	Bremerhaven, Kreisfreie Stadt	0	0	0	0	0	0	0	0	0	0	0
DE600	Hamburg	0	0	0	0	0	0	0	0	0	0	0
DE713	Offenbach am Main, Kreisfreie Stadt	0	0	0	0	0	0	0	0	0	0	0
DE721	Gießen, Landkreis	0	0	0	0	0	0	0	0	0	0	0
DE725	Vogelsbergkreis	0	0	0	0	0	0	0	0	0	0	0
DE731	Kassel, Kreisfreie Stadt	0	0	0	0	0	0	0	0	0	0	0

Code	Name	2012	2013	2014	2015	2016	2017	2018	2019	2020	Cases	NR
DE733	Hersfeld-Rotenburg	0	0	0	0	0	0	0	0	0	0	0
DE734	Kassel, Landkreis	0	0	0	0	0	0	0	0	0	0	0
DE736	Waldeck-Frankenberg	0	0	0	0	0	0	0	0	0	0	0
DE803	Rostock, Kreisfreie Stadt	0	0	0	0	0	0	0	0	0	0	0
DE804	Schwerin, Kreisfreie Stadt	0	0	0	0	0	0	0	0	0	0	0
DE80J	Mecklenburgische Seenplatte	0	0	0	0	0	0	0	0	0	0	0
DE80M	Nordwestmecklenburg	0	0	0	0	0	0	0	0	0	0	0
DE80O	Ludwigslust-Parchim	0	0	0	0	0	0	0	0	0	0	0
DE911	Braunschweig, Kreisfreie Stadt	0	0	0	0	0	0	0	0	0	0	0
DE912	Salzgitter, Kreisfreie Stadt	0	0	0	0	0	0	0	0	0	0	0
DE914	Gifhorn	0	0	0	0	0	0	0	0	0	0	0
DE916	Goslar	0	0	0	0	0	0	0	0	0	0	0
DE918	Northeim	0	0	0	0	0	0	0	0	0	0	0
DE91A	Peine	0	0	0	0	0	0	0	0	0	0	0
DE91B	Wolfenbüttel	0	0	0	0	0	0	0	0	0	0	0
DE922	Diepholz	0	0	0	0	0	0	0	0	0	0	0
DE923	Hameln-Pyrmont	0	0	0	0	0	0	0	0	0	0	0
DE926	Holzminden	0	0	0	0	0	0	0	0	0	0	0
DE928	Schaumburg	0	0	0	0	0	0	0	0	0	0	0
DE933	Harburg	0	0	0	0	0	0	0	0	0	0	0
DE934	Lüchow-Dannenberg	0	0	0	0	0	0	0	0	0	0	0
DE935	Lüneburg, Landkreis	0	0	0	0	0	0	0	0	0	0	0
DE936	Osterholz	0	0	0	0	0	0	0	0	0	0	0

Code	Name	2012	2013	2014	2015	2016	2017	2018	2019	2020	Cases	NR
DE937	Rotenburg (Wümme)	0	0	0	0	0	0	0	0	0	0	0
DE938	Heidekreis	0	0	0	0	0	0	0	0	0	0	0
DE939	Stade	0	0	0	0	0	0	0	0	0	0	0
DE93B	Verden	0	0	0	0	0	0	0	0	0	0	0
DE941	Delmenhorst, Kreisfreie Stadt	0	0	0	0	0	0	0	0	0	0	0
DE942	Emden, Kreisfreie Stadt	0	0	0	0	0	0	0	0	0	0	0
DE943	Oldenburg (Oldenburg), Kreisfreie Stadt	0	0	0	0	0	0	0	0	0	0	0
DE944	Osnabrück, Kreisfreie Stadt	0	0	0	0	0	0	0	0	0	0	0
DE946	Ammerland	0	0	0	0	0	0	0	0	0	0	0
DE947	Aurich	0	0	0	0	0	0	0	0	0	0	0
DE948	Cloppenburg	0	0	0	0	0	0	0	0	0	0	0
DE94A	Friesland (DE)	0	0	0	0	0	0	0	0	0	0	0
DE94B	Grafschaft Bentheim	0	0	0	0	0	0	0	0	0	0	0
DE94C	Leer	0	0	0	0	0	0	0	0	0	0	0
DE94D	Oldenburg, Landkreis	0	0	0	0	0	0	0	0	0	0	0
DE94E	Osnabrück, Landkreis	0	0	0	0	0	0	0	0	0	0	0
DE94F	Vechta	0	0	0	0	0	0	0	0	0	0	0
DE94G	Wesermarsch	0	0	0	0	0	0	0	0	0	0	0
DE94H	Wittmund	0	0	0	0	0	0	0	0	0	0	0
DEA11	Düsseldorf, Kreisfreie Stadt	0	0	0	0	0	0	0	0	0	0	0
DEA13	Essen, Kreisfreie Stadt	0	0	0	0	0	0	0	0	0	0	0
DEA14	Krefeld, Kreisfreie Stadt	0	0	0	0	0	0	0	0	0	0	0
DEA15	Mönchengladbach, Kreisfreie Stadt	0	0	0	0	0	0	0	0	0	0	0

Code	Name	2012	2013	2014	2015	2016	2017	2018	2019	2020	Cases	NR
DEA16	Mülheim an der Ruhr, Kreisfreie Stadt	0	0	0	0	0	0	0	0	0	0	0
DEA17	Oberhausen, Kreisfreie Stadt	0	0	0	0	0	0	0	0	0	0	0
DEA18	Remscheid, Kreisfreie Stadt	0	0	0	0	0	0	0	0	0	0	0
DEA1A	Wuppertal, Kreisfreie Stadt	0	0	0	0	0	0	0	0	0	0	0
DEA1B	Kleve	0	0	0	0	0	0	0	0	0	0	0
DEA1C	Mettmann	0	0	0	0	0	0	0	0	0	0	0
DEA1D	Rhein-Kreis Neuss	0	0	0	0	0	0	0	0	0	0	0
DEA1E	Viersen	0	0	0	0	0	0	0	0	0	0	0
DEA22	Bonn, Kreisfreie Stadt	0	0	0	0	0	0	0	0	0	0	0
DEA23	Köln, Kreisfreie Stadt	0	0	0	0	0	0	0	0	0	0	0
DEA24	Leverkusen, Kreisfreie Stadt	0	0	0	0	0	0	0	0	0	0	0
DEA26	Düren	0	0	0	0	0	0	0	0	0	0	0
DEA29	Heinsberg	0	0	0	0	0	0	0	0	0	0	0
DEA2B	Rheinisch-Bergischer Kreis	0	0	0	0	0	0	0	0	0	0	0
DEA2D	Städteregion Aachen	0	0	0	0	0	0	0	0	0	0	0
DEA31	Bottrop, Kreisfreie Stadt	0	0	0	0	0	0	0	0	0	0	0
DEA32	Gelsenkirchen, Kreisfreie Stadt	0	0	0	0	0	0	0	0	0	0	0
DEA35	Coesfeld	0	0	0	0	0	0	0	0	0	0	0
DEA36	Recklinghausen	0	0	0	0	0	0	0	0	0	0	0
DEA38	Warendorf	0	0	0	0	0	0	0	0	0	0	0
DEA41	Bielefeld, Kreisfreie Stadt	0	0	0	0	0	0	0	0	0	0	0
DEA42	Gütersloh	0	0	0	0	0	0	0	0	0	0	0
DEA43	Herford	0	0	0	0	0	0	0	0	0	0	0

Code	Name	2012	2013	2014	2015	2016	2017	2018	2019	2020	Cases	NR
DEA44	Höxter	0	0	0	0	0	0	0	0	0	0	0
DEA46	Minden-Lübbecke	0	0	0	0	0	0	0	0	0	0	0
DEA51	Bochum, Kreisfreie Stadt	0	0	0	0	0	0	0	0	0	0	0
DEA52	Dortmund, Kreisfreie Stadt	0	0	0	0	0	0	0	0	0	0	0
DEA53	Hagen, Kreisfreie Stadt	0	0	0	0	0	0	0	0	0	0	0
DEA54	Hamm, Kreisfreie Stadt	0	0	0	0	0	0	0	0	0	0	0
DEA55	Herne, Kreisfreie Stadt	0	0	0	0	0	0	0	0	0	0	0
DEA57	Hochsauerlandkreis	0	0	0	0	0	0	0	0	0	0	0
DEA58	Märkischer Kreis	0	0	0	0	0	0	0	0	0	0	0
DEA59	Olpe	0	0	0	0	0	0	0	0	0	0	0
DEA5A	Siegen-Wittgenstein	0	0	0	0	0	0	0	0	0	0	0
DEA5B	Soest	0	0	0	0	0	0	0	0	0	0	0
DEA5C	Unna	0	0	0	0	0	0	0	0	0	0	0
DEB17	Mayen-Koblenz	0	0	0	0	0	0	0	0	0	0	0
DEB18	Neuwied	0	0	0	0	0	0	0	0	0	0	0
DEB1B	Westerwaldkreis	0	0	0	0	0	0	0	0	0	0	0
DEB1C	Cochem-Zell	0	0	0	0	0	0	0	0	0	0	0
DEB1D	Rhein-Hunsrück-Kreis	0	0	0	0	0	0	0	0	0	0	0
DEB21	Trier, Kreisfreie Stadt	0	0	0	0	0	0	0	0	0	0	0
DEB22	Bernkastel-Wittlich	0	0	0	0	0	0	0	0	0	0	0
DEB24	Vulkaneifel	0	0	0	0	0	0	0	0	0	0	0
DEB25	Trier-Saarburg	0	0	0	0	0	0	0	0	0	0	0
DEB31	Frankenthal (Pfalz), Kreisfreie Stadt	0	0	0	0	0	0	0	0	0	0	0

Code	Name	2012	2013	2014	2015	2016	2017	2018	2019	2020	Cases	NR
DEB33	Landau in der Pfalz, Kreisfreie Stadt	0	0	0	0	0	0	0	0	0	0	0
DEB34	Ludwigshafen am Rhein, Kreisfreie Stadt	0	0	0	0	0	0	0	0	0	0	0
DEB35	Mainz, Kreisfreie Stadt	0	0	0	0	0	0	0	0	0	0	0
DEB36	Neustadt an der Weinstraße, Kreisfreie Stadt	0	0	0	0	0	0	0	0	0	0	0
DEB38	Speyer, Kreisfreie Stadt	0	0	0	0	0	0	0	0	0	0	0
DEB39	Worms, Kreisfreie Stadt	0	0	0	0	0	0	0	0	0	0	0
DEB3B	Alzey-Worms	0	0	0	0	0	0	0	0	0	0	0
DEB3D	Donnersbergkreis	0	0	0	0	0	0	0	0	0	0	0
DEB3E	Germersheim	0	0	0	0	0	0	0	0	0	0	0
DEB3G	Kusel	0	0	0	0	0	0	0	0	0	0	0
DEB3H	Südliche Weinstraße	0	0	0	0	0	0	0	0	0	0	0
DEB3I	Rhein-Pfalz-Kreis	0	0	0	0	0	0	0	0	0	0	0
DEB3J	Mainz-Bingen	0	0	0	0	0	0	0	0	0	0	0
DEB3K	Südwestpfalz	0	0	0	0	0	0	0	0	0	0	0
DEC02	Merzig-Wadern	0	0	0	0	0	0	0	0	0	0	0
DEC04	Saarlouis	0	0	0	0	0	0	0	0	0	0	0
DEC06	St. Wendel	0	0	0	0	0	0	0	0	0	0	0
DED51	Leipzig, Kreisfreie Stadt	0	0	0	0	0	0	0	0	0	0	0
DEE04	Altmarkkreis Salzwedel	0	0	0	0	0	0	0	0	0	0	0
DEE07	Börde	0	0	0	0	0	0	0	0	0	0	0
DEE08	Burgenlandkreis	0	0	0	0	0	0	0	0	0	0	0
DEE09	Harz	0	0	0	0	0	0	0	0	0	0	0
DEE0A	Mansfeld-Südharz	0	0	0	0	0	0	0	0	0	0	0

Code	Name	2012	2013	2014	2015	2016	2017	2018	2019	2020	Cases	NR
DEE0C	Salzlandkreis	0	0	0	0	0	0	0	0	0	0	0
DEE0D	Stendal	0	0	0	0	0	0	0	0	0	0	0
DEE0E	Wittenberg	0	0	0	0	0	0	0	0	0	0	0
DEF01	Flensburg, Kreisfreie Stadt	0	0	0	0	0	0	0	0	0	0	0
DEF02	Kiel, Kreisfreie Stadt	0	0	0	0	0	0	0	0	0	0	0
DEF03	Lübeck, Kreisfreie Stadt	0	0	0	0	0	0	0	0	0	0	0
DEF04	Neumünster, Kreisfreie Stadt	0	0	0	0	0	0	0	0	0	0	0
DEF05	Dithmarschen	0	0	0	0	0	0	0	0	0	0	0
DEF06	Herzogtum Lauenburg	0	0	0	0	0	0	0	0	0	0	0
DEF07	Nordfriesland	0	0	0	0	0	0	0	0	0	0	0
DEF08	Ostholstein	0	0	0	0	0	0	0	0	0	0	0
DEF0A	Plön	0	0	0	0	0	0	0	0	0	0	0
DEF0B	Rendsburg-Eckernförde	0	0	0	0	0	0	0	0	0	0	0
DEF0C	Schleswig-Flensburg	0	0	0	0	0	0	0	0	0	0	0
DEF0D	Segeberg	0	0	0	0	0	0	0	0	0	0	0
DEF0E	Steinburg	0	0	0	0	0	0	0	0	0	0	0
DEF0F	Stormarn	0	0	0	0	0	0	0	0	0	0	0
DEG01	Erfurt, Kreisfreie Stadt	0	0	0	0	0	0	0	0	0	0	0
DEG07	Nordhausen	0	0	0	0	0	0	0	0	0	0	0
DEG09	Unstrut-Hainich-Kreis	0	0	0	0	0	0	0	0	0	0	0
DEG0A	Kyffhäuserkreis	0	0	0	0	0	0	0	0	0	0	0
DEG0C	Gotha	0	0	0	0	0	0	0	0	0	0	0
DEG0D	Sömmerda	0	0	0	0	0	0	0	0	0	0	0

Code	Name	2012	2013	2014	2015	2016	2017	2018	2019	2020	Cases	NR
DEG0M	Altenburger Land	0	0	0	0	0	0	0	0	0	0	0
DEG0N	Eisenach, Kreisfreie Stadt	0	0	0	0	0	0	0	0	0	0	0
DEG0P	Wartburgkreis	0	0	0	0	0	0	0	0	0	0	0
**EL**	**GREECE**											
EL515	Θάσος, Καβάλα	0	0	0.7	0	0	0	0	0	0	1	0.1
EL651	Αργολίδα, Αρκαδία	0	0	0	0.6	0	0	0	0	0	1	0.1
EL301	Βόρειος Τομέας Αθηνών	0	0	0	0	0	0	0	0	0	0	0
EL302	Δυτικός Τομέας Αθηνών	0	0	0	0	0	0	0	0	0	0	0
EL303	Κεντρικός Τομέας Αθηνών	0	0	0	0	0	0	0	0	0	0	0
EL304	Νότιος Τομέας Αθηνών	0	0	0	0	0	0	0	0	0	0	0
EL305	Ανατολική Αττική	0	0	0	0	0	0	0	0	0	0	0
EL306	Δυτική Αττική	0	0	0	0	0	0	0	0	0	0	0
EL307	Πειραιάς, Νήσοι	0	0	0	0	0	0	0	0	0	0	0
EL411	Λέσβος, Λήμνος	0	0	0	0	0	0	0	0	0	0	0
EL412	Ικαρία, Σάμος	0	0	0	0	0	0	0	0	0	0	0
EL413	Χίος	0	0	0	0	0	0	0	0	0	0	0
EL421	Κάλυμνος, Κάρπαθος – Ηρωική Νήσος Κάσος, Κως, Ρόδος	0	0	0	0	0	0	0	0	0	0	0
EL422	Άνδρος, Θήρα, Κέα, Μήλος, Μύκονος, Νάξος, Πάρος, Σύρος, Τήνος	0	0	0	0	0	0	0	0	0	0	0
EL431	Ηράκλειο	0	0	0	0	0	0	0	0	0	0	0
EL432	Λασίθι	0	0	0	0	0	0	0	0	0	0	0
EL433	Ρέθυμνο	0	0	0	0	0	0	0	0	0	0	0
EL434	Χανιά	0	0	0	0	0	0	0	0	0	0	0
EL511	Έβρος	0	0	0	0	0	0	0	0	0	0	0

Code	Name	2012	2013	2014	2015	2016	2017	2018	2019	2020	Cases	NR
EL512	Ξάνθη	0	0	0	0	0	0	0	0	0	0	0
EL513	Ροδόπη	0	0	0	0	0	0	0	0	0	0	0
EL514	Δράμα	0	0	0	0	0	0	0	0	0	0	0
EL521	Ημαθία	0	0	0	0	0	0	0	0	0	0	0
EL522	Θεσσαλονίκη	0	0	0	0	0	0	0	0	0	0	0
EL523	Κιλκίς	0	0	0	0	0	0	0	0	0	0	0
EL524	Πέλλα	0	0	0	0	0	0	0	0	0	0	0
EL525	Πιερία	0	0	0	0	0	0	0	0	0	0	0
EL526	Σέρρες	0	0	0	0	0	0	0	0	0	0	0
EL527	Χαλκιδική	0	0	0	0	0	0	0	0	0	0	0
EL531	Γρεβενά, Κοζάνη	0	0	0	0	0	0	0	0	0	0	0
EL532	Καστοριά	0	0	0	0	0	0	0	0	0	0	0
EL533	Φλώρινα	0	0	0	0	0	0	0	0	0	0	0
EL541	Άρτα, Πρέβεζα	0	0	0	0	0	0	0	0	0	0	0
EL542	Θεσπρωτία	0	0	0	0	0	0	0	0	0	0	0
EL543	Ιωάννινα	0	0	0	0	0	0	0	0	0	0	0
EL611	Καρδίτσα, Τρίκαλα	0	0	0	0	0	0	0	0	0	0	0
EL612	Λάρισα	0	0	0	0	0	0	0	0	0	0	0
EL613	Μαγνησία, Σποράδες	0	0	0	0	0	0	0	0	0	0	0
EL621	Ζάκυνθος	0	0	0	0	0	0	0	0	0	0	0
EL622	Κέρκυρα	0	0	0	0	0	0	0	0	0	0	0
EL623	Ιθάκη, Κεφαλληνία	0	0	0	0	0	0	0	0	0	0	0
EL624	Λευκάδα	0	0	0	0	0	0	0	0	0	0	0

Code	Name	2012	2013	2014	2015	2016	2017	2018	2019	2020	Cases	NR
EL631	Αιτωλοακαρνανία	0	0	0	0	0	0	0	0	0	0	0
EL632	Αχαΐα	0	0	0	0	0	0	0	0	0	0	0
EL633	Ηλεία	0	0	0	0	0	0	0	0	0	0	0
EL641	Βοιωτία	0	0	0	0	0	0	0	0	0	0	0
EL642	Εὺβοια	0	0	0	0	0	0	0	0	0	0	0
EL643	Ευρυτανία	0	0	0	0	0	0	0	0	0	0	0
EL644	Φθιώτιδα	0	0	0	0	0	0	0	0	0	0	0
EL645	Φωκίδα	0	0	0	0	0	0	0	0	0	0	0
EL652	Κορινθία	0	0	0	0	0	0	0	0	0	0	0
EL653	Λακωνία, Μεσσηνία	0	0	0	0	0	0	0	0	0	0	0
**HU**	**HUNGARY**											
HU223	Zala	2.1	5	1.4	0.4	1.4	0.7	2.6	1.1	1.1	44	1.8
HU222	Vas	2	2.7	2.4	0.8	2	0	2.8	0.4	0.8	35	1.5
HU232	Somogy	1.6	2.8	1.3	1.3	0.3	1	0.7	0	1	31	1.1
HU313	Nógrád	2	1	0.5	0.5	1	0	1	0	0	12	0.7
HU211	Fejér	0	0.7	0.2	0.2	1	0.7	1.2	0.5	0.5	21	0.6
HU233	Tolna	0.9	0.4	0	1.3	0.4	0.4	0	0	0	8	0.4
HU312	Heves	0.7	0.3	0.3	0.7	0	0	0	0.3	1.4	11	0.4
HU231	Baranya	0.5	0.3	0.5	0.3	0	0.3	0	0.3	0.3	9	0.3
HU213	Veszprém	0.3	0.3	0.6	0	0	0.3	0	0.6	0.3	8	0.3
HU311	Borsod-Abaúj-Zemplén	0.6	0.4	0.3	0.1	0	0	0.1	0.2	0.2	13	0.2
HU221	Győr-Moson-Sopron	0.9	0	0.2	0.4	0	0	0.2	0	0	8	0.2
HU333	Csongrád	0.2	0.2	0	0.2	0	0	0.2	0	0	4	0.1

Code	Name	2012	2013	2014	2015	2016	2017	2018	2019	2020	Cases	NR
HU120	Pest	0.5	0.1	0.3	0.1	0.1	0.2	0	0.1	0.1	17	0.1
HU110	Budapest	0.1	0.1	0.1	0.2	0.1	0.1	0.1	0.2	0	15	0.1
HU332	Békés	0.3	0.3	0	0	0	0	0.3	0	0	3	0.1
HU321	Hajdú-Bihar	0	0.4	0	0	0	0	0.2	0.2	0	4	0.1
HU331	Bács-Kiskun	0	0.2	0	0.2	0	0.2	0.2	0	0	4	0.1
HU212	Komárom-Esztergom	0	0	0.3	0	0	0	0	0.7	0	3	0.1
HU322	Jász-Nagykun-Szolnok	0	0	0	0	0	0	0	0	0	0	0
HU323	Szabolcs-Szatmár-Bereg	0	0	0	0	0	0	0	0	0	0	0
**IE**	**IRELAND**											
IE041	Border	0	0	0	0	0	0	0	0	0	0	0
IE042	West	0	0	0	0	0	0	0	0	0	0	0
IE051	Mid-West	0	0	0	0	0	0	0	0	0	0	0
IE052	South-East	0	0	0	0	0	0	0	0	0	0	0
IE053	South-West	0	0	0	0	0	0	0	0	0	0	0
IE061	Dublin	0	0	0	0	0	0	0	0	0	0	0
IE062	Mid-East	0	0	0	0	0	0	0	0	0	0	0
IE063	Midland	0	0	0	0	0	0	0	0	0	0	0
**LV**	**LATVIA**											
LV003	Kurzeme	12.4	25.9	18.6	23.2	39.8	36.1	30	32.9	33.7	629	27.8
LV007	Pierīga	14.3	15.9	8.7	7.6	8.5	12.9	0.8	16.7	12.3	361	10.9
LV008	Vidzeme	17.2	16	8.9	5.5	4.6	6.3	7.4	5.9	4.9	153	8.7
LV009	Zemgale	8	10.4	5.3	3.7	7.1	8.5	3	8.7	0	132	6.1
LV005	Latgale	16.1	5.1	3.8	4.6	5.1	6.7	1.5	1.5	1.2	130	5.2

Code	Name	2012	2013	2014	2015	2016	2017	2018	2019	2020	Cases	NR
LV006	Rīga	5.8	4.7	4	3.4	5.2	4.7	5.6	4.7	6.5	286	5
**LT**	**LITHUANIA**											
LT029	Utenos apskritis	43	35	44.9	47.1	53.9	43.5	34.7	55.9	62.4	571	46.4
LT021	Alytaus apskritis	20	24.3	24.7	15.6	29.6	26.1	13.8	14.7	14.2	266	20.5
LT025	Panevėžio apskritis	22.3	20.6	16.3	14.4	26.4	12.4	18.3	20	27.9	409	19.8
LT022	Kauno apskritis	12	15.2	10.4	12.5	23.6	16	13.8	21.7	22.2	848	16.3
LT011	Vilniaus apskritis	10.2	11.9	8.3	9	15.3	15.2	12.2	18.8	31	1067	14.7
LT026	Šiaulių apskritis	13.5	14.8	9.8	9.9	23.5	13.3	11.3	19.8	13.8	358	14.4
LT024	Marijampolės apskritis	3.8	3.2	6.5	5.9	30.9	17.9	13.4	30.3	16.1	185	13.9
LT027	Tauragės apskritis	4.6	2.8	5.7	1.9	23.7	17.2	8.3	12.8	14.2	90	10
LT023	Klaipėdos apskritis	4.5	9.3	8.5	6.1	12.3	11.5	9.8	11.3	16.9	292	10
LT028	Telšių apskritis	4	3.4	6.2	2.1	12.7	9.4	8.9	18.9	14.6	110	8.7
**PL**	**POLAND**											
PL841	Białostocki	10.9	12.1	12.3	7.8	17.5	13.3	8.5	8.1	1.4	463	10.2
PL843	Suwalski	11.2	12.7	10.4	6.4	13.2	17.7	4.9	8.3	3	234	9.8
PL842	Łomżyński	4.6	4.3	4.6	5.6	10	10.8	5.7	10.9	7.1	247	7
PL623	Ełcki	3.9	7.4	3.5	3.2	10.3	8.5	5.7	5	3.2	143	5.6
PL622	Olsztyński	3.4	5	2.8	2.5	2.5	3.1	1.5	5	0.3	159	2.9
PL517	Wałbrzyski	0.7	0.8	0.8	0.9	1.4	1.5	1.9	2.8	1.7	81	1.4
PL924	Ostrołęcki	1.3	1.6	1.3	1.3	1.8	1.6	1.8	0.8	0	44	1.3
PL811	Bialski	0.3	1	1.6	0.7	2	1.3	2	1.7	1.4	36	1.3
PL925	Siedlecki	1.2	0.5	0.9	0.2	1.7	1.7	1.9	1	0.7	41	1.1
PL722	Sandomiersko-jędrzejowski	0	0	0.2	0.4	1	1	1.3	1.5	3	40	0.9

Code	Name	2012	2013	2014	2015	2016	2017	2018	2019	2020	Cases	NR
PL523	Nyski	1.1	0.8	0.8	0.8	1.1	0.3	0.8	0.6	0	23	0.7
PL621	Elbląski	0.8	0.4	0.6	0.4	0.8	1.1	0.4	0.4	1.4	32	0.7
PL524	Opolski	0.7	0.5	0.5	0	0.5	0.2	0.9	0.2	0	20	0.4
PL913	Warszawski zachodni	0.2	0.2	0.2	0.2	0.3	0	0.2	0.3	2.3	24	0.4
PL214	Krakowski	0	0.3	0.1	0	0.3	0.7	1.8	0.7	0.1	29	0.4
PL516	Legnicko-głogowski	0	0.4	0	0	0	0.4	0.2	0.4	2	16	0.4
PL213	Miasto Kraków	0.3	0.5	0	0.7	0	0.1	0.7	0.4	0	20	0.3
PL912	Warszawski wschodni	0.2	0	0.2	0	0.6	0.2	0.3	0.6	0.6	17	0.3
PL515	Jeleniogórski	0.2	0.3	0.5	0	0.2	0.4	0.5	0.2	0.6	16	0.3
PL518	Wrocławski	0	0.5	0.2	0	0.5	0.2	0.3	0.7	0.5	17	0.3
PL815	Puławski	0	0.2	0.2	0.4	0	0.8	0.6	0.8	0	15	0.3
PL921	Radomski	0	0	0	0	0	0.2	0	0.5	1.8	15	0.3
PL514	Miasto Wrocław	0.5	0.3	0.2	0.3	0.2	0.3	0	0.2	0	12	0.2
PL714	Sieradzki	0.2	0	0	0	0.2	0.4	0.4	0.9	0	10	0.2
PL922	Ciechanowski	0.3	0	0.6	0	0.3	0	0	0	0.6	6	0.2
PL911	Miasto Warszawa	0.1	0.4	0.3	0.5	0.1	0.3	0.2	0.2	0	36	0.2
PL21A	Oświęcimski	0.2	0.4	0.2	0	0.2	0.4	0.2	0.2	0.2	10	0.2
PL721	Kielecki	0.1	0.1	0.4	0	0.3	0.4	0.4	0.4	0.1	17	0.2
PL711	Miasto Łódź	0.1	0	0.1	0.3	0.1	0.6	0.3	0.6	0	15	0.2
PL712	Łódzki	0.3	0.5	0	0	0.3	0.3	0.5	0	0	7	0.2
PL217	Tarnowski	0	0.2	0.2	0	0	0.7	0	0.4	0	7	0.2
PL418	Poznański	0.2	0	0.2	0.3	0	0.2	0	0	0.1	6	0.1
PL22B	Sosnowiecki	0.1	0	0.3	0	0	0	0.1	0.1	0	5	0.1

Code	Name	2012	2013	2014	2015	2016	2017	2018	2019	2020	Cases	NR
PL424	Miasto Szczecin	0.2	0	0	0.2	0.2	0	0	0.2	0	4	0.1
PL923	Płocki	0	0.3	0	0	0	0	0	0.3	0	2	0.1
PL812	Chełmsko-zamojski	0	0.2	0	0	0	0.3	0.3	0.2	0	6	0.1
PL824	Tarnobrzeski	0	0.2	0	0.2	0.2	0.2	0.3	0.2	0	7	0.1
PL613	Bydgosko-toruński	0	0	0.1	0.1	0	0	0	0.3	0	4	0.1
PL634	Gdański	0	0	0.2	0.2	0.2	0.2	0	0	0	4	0.1
PL713	Piotrkowski	0	0	0	0.2	0.2	0	0	0.2	0	3	0.1
PL618	Świecki	0	0	0	0	0.5	0	0	0	0	1	0.1
PL715	Skierniewicki	0	0	0	0	0.3	0	0.3	0	0	2	0.1
PL814	Lubelski	0	0	0	0	0	0.1	0.3	0.3	0.1	6	0.1
PL426	Koszaliński	0	0	0	0	0	0	0	0.6	0	2	0.1
PL416	Kaliski	0.1	0	0	0	0	0	0	0.1	0	2	0
PL414	Koniński	0.1	0	0.1	0	0	0	0	0	0	2	0
PL229	Gliwicki	0.2	0	0	0.2	0	0	0	0	0	2	0
PL823	Rzeszowski	0.2	0	0	0	0	0.2	0	0	0	2	0
PL415	Miasto Poznań	0.2	0	0	0	0.2	0	0	0	0	2	0
PL638	Starogardzki	0	0.2	0	0	0	0	0	0.2	0	2	0
PL225	Bielski	0	0.1	0	0	0	0	0	0	0	1	0
PL428	Szczeciński	0	0.2	0	0	0	0	0.2	0	0	2	0
PL431	Gorzowski	0	0	0	0	0.3	0	0	0	0	1	0
PL218	Nowosądecki	0	0	0	0	0.2	0	0	0	0	1	0
PL633	Trójmiejski	0	0	0	0	0.1	0	0	0	0	1	0
PL821	Krośnieński	0	0	0	0	0.2	0	0	0	0	1	0

Code	Name	2012	2013	2014	2015	2016	2017	2018	2019	2020	Cases	NR
PL427	Szczecinecko-pyrzycki	0	0	0	0	0	0.2	0	0.2	0	2	0
PL224	Częstochowski	0	0	0	0	0	0	0	0.2	0	1	0
PL617	Inowrocławski	0	0	0	0	0	0	0	0.3	0	1	0
PL227	Rybnicki	0	0	0	0	0	0	0	0.3	0	2	0
PL637	Chojnicki	0	0	0	0	0	0	0	0	0.4	1	0
PL219	Nowotarski	0	0	0	0	0	0	0	0	0	0	0
PL228	Bytomski	0	0	0	0	0	0	0	0	0	0	0
PL22A	Katowicki	0	0	0	0	0	0	0	0	0	0	0
PL22C	Tyski	0	0	0	0	0	0	0	0	0	0	0
PL411	Pilski	0	0	0	0	0	0	0	0	0	0	0
PL417	Leszczyński	0	0	0	0	0	0	0	0	0	0	0
PL432	Zielonogórski	0	0	0	0	0	0	0	0	0	0	0
PL616	Grudziądzki	0	0	0	0	0	0	0	0	0	0	0
PL619	Włocławski	0	0	0	0	0	0	0	0	0	0	0
PL636	Słupski	0	0	0	0	0	0	0	0	0	0	0
PL822	Przemyski	0	0	0	0	0	0	0	0	0	0	0
PL926	Żyrardowski	0	0	0	0	0	0	0	0	0	0	0
**RO**	**ROMANIA**											
RO126	Sibiu	0.8	0	0.2	0	0	0	0	0	0	4	0.1
RO125	Mureş	0	0.6	0	0	0	0	0.4	0	0	5	0.1
RO111	Bihor	0	0	0	0	0	0	0	0	0	0	0
RO112	Bistriţa-Năsăud	0	0	0	0	0	0	0	0	0	0	0
RO113	Cluj	0	0	0	0	0	0	0	0	0	0	0

Code	Name	2012	2013	2014	2015	2016	2017	2018	2019	2020	Cases	NR
RO114	Maramureş	0	0	0	0	0	0	0	0	0	0	0
RO115	Satu Mare	0	0	0	0	0	0	0	0	0	0	0
RO116	Sălaj	0	0	0	0	0	0	0	0	0	0	0
RO121	Alba	0	0	0	0	0	0	0	0	0	0	0
RO122	Braşov	0	0	0	0	0	0	0	0	0	0	0
RO123	Covasna	0	0	0	0	0	0	0	0	0	0	0
RO124	Harghita	0	0	0	0	0	0	0	0	0	0	0
RO211	Bacău	0	0	0	0	0	0	0	0	0	0	0
RO212	Botoşani	0	0	0	0	0	0	0	0	0	0	0
RO213	Iaşi	0	0	0	0	0	0	0	0	0	0	0
RO214	Neamţ	0	0	0	0	0	0	0	0	0	0	0
RO215	Suceava	0	0	0	0	0	0	0	0	0	0	0
RO216	Vaslui	0	0	0	0	0	0	0	0	0	0	0
RO221	Brăila	0	0	0	0	0	0	0	0	0	0	0
RO222	Buzău	0	0	0	0	0	0	0	0	0	0	0
RO223	Constanţa	0	0	0	0	0	0	0	0	0	0	0
RO224	Galaţi	0	0	0	0	0	0	0	0	0	0	0
RO225	Tulcea	0	0	0	0	0	0	0	0	0	0	0
RO226	Vrancea	0	0	0	0	0	0	0	0	0	0	0
RO311	Argeş	0	0	0	0	0	0	0	0	0	0	0
RO312	Călăraşi	0	0	0	0	0	0	0	0	0	0	0
RO313	Dâmboviţa	0	0	0	0	0	0	0	0	0	0	0
RO314	Giurgiu	0	0	0	0	0	0	0	0	0	0	0

Code	Name	2012	2013	2014	2015	2016	2017	2018	2019	2020	Cases	NR
RO315	Ialomiţa	0	0	0	0	0	0	0	0	0	0	0
RO316	Prahova	0	0	0	0	0	0	0	0	0	0	0
RO317	Teleorman	0	0	0	0	0	0	0	0	0	0	0
RO321	Bucureşti	0	0	0	0	0	0	0	0	0	0	0
RO322	Ilfov	0	0	0	0	0	0	0	0	0	0	0
RO411	Dolj	0	0	0	0	0	0	0	0	0	0	0
RO412	Gorj	0	0	0	0	0	0	0	0	0	0	0
RO413	Mehedinţi	0	0	0	0	0	0	0	0	0	0	0
RO414	Olt	0	0	0	0	0	0	0	0	0	0	0
RO415	Vâlcea	0	0	0	0	0	0	0	0	0	0	0
RO421	Arad	0	0	0	0	0	0	0	0	0	0	0
RO422	Caraş-Severin	0	0	0	0	0	0	0	0	0	0	0
RO423	Hunedoara	0	0	0	0	0	0	0	0	0	0	0
RO424	Timiş	0	0	0	0	0	0	0	0	0	0	0
**SK**	**SLOVAKIA**											
SK032	Banskobystrický kraj	3	3.5	2	3.5	9.3	3.1	7.2	6.2	11.5	321	5.5
SK031	Žilinský kraj	4.5	6.1	5.1	3.8	3.3	2.8	5.6	6.8	8.4	320	5.2
SK022	Trenčiansky kraj	4.5	11.3	5.6	2	3.6	1.5	5.6	5.6	5.7	268	5
SK023	Nitriansky kraj	1	1.7	1.9	1.8	1.5	0.7	1.5	2.2	0.6	88	1.4
SK042	Košický kraj	0.6	0.5	0.4	0.8	6	0.8	0.6	0.5	0.4	84	1.2
SK041	Prešovský kraj	0.5	1.1	1.1	0.5	0.4	1.7	1.9	1.4	1.1	80	1.1
SK021	Trnavský kraj	0.7	0.9	0.7	0.2	0.5	0.5	0.7	0	0.2	25	0.5
SK010	Bratislavský kraj	0.5	0.2	0.8	0	0.3	0	0.5	1.1	0.3	23	0.4

Code	Name	2012	2013	2014	2015	2016	2017	2018	2019	2020	Cases	NR
**SL**	**SLOVENIA**											
SI033	Koroška	13.8	30.5	12.6	8.4	18.3	18.4	14.2	11.3	36.8	117	18.2
SI042	Gorenjska	17.6	39.2	8.3	4.4	5.4	9.8	20.1	11.2	20.8	280	15.2
SI038	Primorsko-notranjska	21	0	0	1.9	11.4	13.3	13.4	20.9	34.1	61	12.9
SI041	Osrednjeslovenska	5.6	23.4	6.4	3.5	4.3	5.2	8.5	6	6.8	373	7.7
SI034	Savinjska	12.6	13.4	5.9	3.1	2	3.1	5.1	3.5	4.7	136	5.9
SI037	Jugovzhodna Slovenija	6.3	4.2	1.4	4.2	2.8	2.1	6.3	5.6	11	63	4.9
SI031	Pomurska	4.2	6.8	4.3	1.7	3.5	4.3	4.4	4.4	5.2	45	4.3
SI032	Podravska	6.2	6.8	4	2.5	3.7	3.7	5	2.8	3.4	123	4.2
SI043	Goriška	4.2	5.9	0	0.8	3.4	2.5	4.3	2.5	11.9	42	4
SI044	Obalno-kraška	0.9	4.5	4.4	0	0	0.9	0.9	0	0.9	14	1.4
SI036	Posavska	6.6	0	0	0	1.3	2.6	0	1.3	0	9	1.3
SI035	Zasavska	1.7	0	0	3.5	0	0	0	1.8	3.5	6	1.2
**ES**	**SPAIN**											
ES111	A Coruña	0	0	0	0	0	0	0	0	0	0	0
ES112	Lugo	0	0	0	0	0	0	0	0	0	0	0
ES113	Ourense	0	0	0	0	0	0	0	0	0	0	0
ES114	Pontevedra	0	0	0	0	0	0	0	0	0	0	0
ES120	Asturias	0	0	0	0	0	0	0	0	0	0	0
ES130	Cantabria	0	0	0	0	0	0	0	0	0	0	0
ES211	Araba/Álava	0	0	0	0	0	0	0	0	0	0	0
ES212	Gipuzkoa	0	0	0	0	0	0	0	0	0	0	0
ES213	Bizkaia	0	0	0	0	0	0	0	0	0	0	0

Code	Name	2012	2013	2014	2015	2016	2017	2018	2019	2020	Cases	NR
ES220	Navarra	0	0	0	0	0	0	0	0	0	0	0
ES230	La Rioja	0	0	0	0	0	0	0	0	0	0	0
ES241	Huesca	0	0	0	0	0	0	0	0	0	0	0
ES242	Teruel	0	0	0	0	0	0	0	0	0	0	0
ES243	Zaragoza	0	0	0	0	0	0	0	0	0	0	0
ES300	Madrid	0	0	0	0	0	0	0	0	0	0	0
ES411	Ávila	0	0	0	0	0	0	0	0	0	0	0
ES412	Burgos	0	0	0	0	0	0	0	0	0	0	0
ES413	León	0	0	0	0	0	0	0	0	0	0	0
ES414	Palencia	0	0	0	0	0	0	0	0	0	0	0
ES415	Salamanca	0	0	0	0	0	0	0	0	0	0	0
ES416	Segovia	0	0	0	0	0	0	0	0	0	0	0
ES417	Soria	0	0	0	0	0	0	0	0	0	0	0
ES418	Valladolid	0	0	0	0	0	0	0	0	0	0	0
ES419	Zamora	0	0	0	0	0	0	0	0	0	0	0
ES421	Albacete	0	0	0	0	0	0	0	0	0	0	0
ES422	Ciudad Real	0	0	0	0	0	0	0	0	0	0	0
ES423	Cuenca	0	0	0	0	0	0	0	0	0	0	0
ES424	Guadalajara	0	0	0	0	0	0	0	0	0	0	0
ES425	Toledo	0	0	0	0	0	0	0	0	0	0	0
ES431	Badajoz	0	0	0	0	0	0	0	0	0	0	0
ES432	Cáceres	0	0	0	0	0	0	0	0	0	0	0
ES511	Barcelona	0	0	0	0	0	0	0	0	0	0	0

Code	Name	2012	2013	2014	2015	2016	2017	2018	2019	2020	Cases	NR
ES512	Girona	0	0	0	0	0	0	0	0	0	0	0
ES513	Lleida	0	0	0	0	0	0	0	0	0	0	0
ES514	Tarragona	0	0	0	0	0	0	0	0	0	0	0
ES521	Alicante/Alacant	0	0	0	0	0	0	0	0	0	0	0
ES522	Castellón/Castelló	0	0	0	0	0	0	0	0	0	0	0
ES523	Valencia/València	0	0	0	0	0	0	0	0	0	0	0
ES531	Eivissa y Formentera	0	0	0	0	0	0	0	0	0	0	0
ES532	Mallorca	0	0	0	0	0	0	0	0	0	0	0
ES533	Menorca	0	0	0	0	0	0	0	0	0	0	0
ES611	Almería	0	0	0	0	0	0	0	0	0	0	0
ES612	Cádiz	0	0	0	0	0	0	0	0	0	0	0
ES613	Córdoba	0	0	0	0	0	0	0	0	0	0	0
ES614	Granada	0	0	0	0	0	0	0	0	0	0	0
ES615	Huelva	0	0	0	0	0	0	0	0	0	0	0
ES616	Jaén	0	0	0	0	0	0	0	0	0	0	0
ES617	Málaga	0	0	0	0	0	0	0	0	0	0	0
ES618	Sevilla	0	0	0	0	0	0	0	0	0	0	0
ES620	Murcia	0	0	0	0	0	0	0	0	0	0	0
ES630	Ceuta	0	0	0	0	0	0	0	0	0	0	0
ES640	Melilla	0	0	0	0	0	0	0	0	0	0	0
ES703	El Hierro	0	0	0	0	0	0	0	0	0	0	0
ES704	Fuerteventura	0	0	0	0	0	0	0	0	0	0	0
ES705	Gran Canaria	0	0	0	0	0	0	0	0	0	0	0

Code	Name	2012	2013	2014	2015	2016	2017	2018	2019	2020	Cases	NR
ES706	La Gomera	0	0	0	0	0	0	0	0	0	0	0
ES707	La Palma	0	0	0	0	0	0	0	0	0	0	0
ES708	Lanzarote	0	0	0	0	0	0	0	0	0	0	0
ES709	Tenerife	0	0	0	0	0	0	0	0	0	0	0
**SE**	**SWEDEN**											
SE122	Södermanlands län	18	11.3	7.2	14.2	7.8	12.5	16.5	8.5	6.4	290	11.3
SE121	Uppsala län	13.3	5.6	7	14.9	12.1	14.4	12.2	9.3	7.8	345	10.7
SE125	Västmanlands län	0.8	2.7	1.5	6.9	6	4.9	8.5	8.4	7.2	126	5.3
SE110	Stockholms län	6.6	4.4	3.9	3.5	3.6	6.2	4.7	4	2.7	880	4.4
SE123	Östergötlands län	2.1	3.5	2.3	1.4	2.9	4.4	3.7	6.9	3.9	140	3.5
SE311	Värmlands län	1.5	1.1	0.7	1.5	2.2	5	4.6	6.8	4.2	77	3.1
SE232	Västra Götalands län	1.3	1.1	1.2	2.5	1.6	3.6	3.5	3.8	3	363	2.4
SE124	Örebro län	1.4	0.3	0	1.7	2.4	2	5.3	2.6	5.2	63	2.4
SE214	Gotlands län	1.7	0	0	7	0	5.2	3.4	3.4	0	12	2.3
SE221	Blekinge län	0.7	1.3	0	0	0.6	5.7	3.1	1.9	3.1	26	1.9
SE211	Jönköpings län	0.3	1.2	1.2	0.6	0.3	2.3	4.2	1.9	1.6	48	1.5
SE213	Kalmar län	0.9	0.9	1.3	0	1.7	3.3	0.4	2.5	1.6	30	1.4
SE212	Kronobergs län	1.6	1.1	0	1.6	1	1	2.5	1.5	0.5	21	1.2
SE313	Gävleborgs län	0	0.7	0.4	0.4	0.7	0	0.3	2.4	1	17	0.7
SE312	Dalarnas län	0.4	0	0.4	0	0.7	0.3	1	2.4	0	15	0.6
SE224	Skåne län	0.2	0.6	0.2	0.4	0.5	0.6	0.5	0.7	0.7	57	0.5
SE231	Hallands län	0	0	0	0	0	0	1.2	0.9	0.9	10	0.3
SE321	Västernorrlands län	0	0	0	0	0	0	0	0	0	0	0

Code	Name	2012	2013	2014	2015	2016	2017	2018	2019	2020	Cases	NR
SE322	Jämtlands län	0	0	0	0	0	0	0	0	0	0	0
SE331	Västerbottens län	0	0	0	0	0	0	0	0	0	0	0
SE332	Norrbottens län	0	0	0	0	0	0	0	0	0	0	0

Legend: Code (NUTS code); Name (name of region); Cases (total number of cases in period 2012-2020); NR (average notification rate in period 2012-2020)

Supplementary material S6. Tick-borne encephalitis vaccination recommendations in high endemic regions and cost coverage

High-endemic regions (i.e., notification rate ≥ 5 / 100,000) were found in ten countries: Czechia, Estonia, Germany, Finland, Latvia, Lithuania, Poland, Slovakia, Slovenia and Sweden. Co-authors from the respective countries and country representatives of Estonia and Latvia were asked to complete the table below. Information about TBE recommendation in Latvia was extracted elsewhere [1].

Supplementary Table S6. Tick-borne encephalitis vaccination recommendations in high endemic regions and cost coverage

Code	Name	Notification rate	Recommendation	Free of charge
		**Czechia**		
**CZ031**	Jihočeský kraj	18.0	Yes	Partially
**CZ063**	Kraj Vysočina	14.2	Yes	Partially
**CZ032**	Plzeňský kraj	8.1	Yes	Partially
**CZ053**	Pardubický kraj	7.4	Yes	Partially
**CZ071**	Olomoucký kraj	7.1	Yes	Partially
**CZ041**	Karlovarský kraj	5.9	Yes	Partially
**CZ072**	Zlínský kraj	5.8	Yes	Partially
**CZ042**	Ústecký kraj	5.0	Yes	Partially
		**Estonia**		
**EE004**	Lääne-Eesti	28.0	Unknown	Unknown
**EE008**	Lõuna-Eesti	7.5	Unknown	Unknown
**EE009**	Kesk-Eesti	5.0	Unknown	Unknown
		**Germany**		
**DE234**	Amberg-Sulzbach	9.7	Yes	Yes
**DE12A**	Calw	7.0	Yes	Yes
**DE239**	Schwandorf	6.5	Yes	Yes
**DE225**	Freyung-Grafenau	6.4	Yes	Yes
**DE258**	Fürth, Landkreis	5.9	Yes	Yes
**DE12C**	Freudenstadt	5.7	Yes	Yes
**DE237**	Neustadt a. d. Waldnaab	5.5	Yes	Yes
		**Finland**		
**FI200**	Åland	26.4	Unknown	Unknown
		**Latvia**		
**LV003**	Kurzeme	27.8	Yes	Unknown
**LV007**	Pierīga	10.9	Yes	Unknown
**LV008**	Vidzeme	8.7	Yes	Unknown
**LV009**	Zemgale	6.1	Yes	Unknown
**LV005**	Latgale	5.2	Yes	Unknown
**LV006**	Rīga	5.0	Yes	Unknown
		**Lithuania**		
**LT029**	Utenos apskritis	46.4	Yes	No
**LT021**	Alytaus apskritis	20.5	Yes	No
**LT025**	Panevėžio apskritis	19.8	Yes	No
**LT022**	Kauno apskritis	16.3	Yes	No
**LT011**	Vilniaus apskritis	14.7	Yes	No

**LT026**	Šiaulių apskritis	14.4	Yes	No
**LT024**	Marijampolės apskritis	13.9	Yes	No
**LT027**	Tauragės apskritis	10.0	Yes	No
**LT023**	Klaipėdos apskritis	10.0	Yes	No
**LT028**	Telšių apskritis	8.7	Yes	No
		**Poland**		
**PL841**	Białostocki	10.2	Yes	Partially
**PL843**	Suwalski	9.8	Yes	Partially
**PL842**	Łomżyński	7.0	Yes	Partially
**PL623**	Ełcki	5.6	Yes	Partially
		**Slovakia**		
**SK032**	Banskobystrický kraj	5.5	No	No
**SK031**	Žilinský kraj	5.2	No	No
**SK022**	Trenčiansky kraj	5.0	No	No
		**Slovenia**		
**SI033**	Koroška	18.2	Yes	Partially
**SI042**	Gorenjska	15.2	Yes	Partially
**SI038**	Primorsko-notranjska	12.9	Yes	Partially
**SI041**	Osrednjeslovenska	7.7	Yes	Partially
**SI034**	Savinjska	5.9	Yes	Partially
		**Sweden**		
**SE122**	Södermanlands län	11.3	Yes	Partially
**SE121**	Uppsala län	10.7	Yes	Partially
**SE125**	Västmanlands län	5.3	Yes	No

Legend: Code (NUTS code); Name (name of region); Recommendation (recommendation for TBE vaccination for all inhabitants in the specific region by the national/regional health authorities); Free of charge (vaccination against tick-borne encephalitis is free of charge for all inhabitants of the specific region).

Reference:

1. Zavadska D, Odzelevica Z, Karelis G, Liepina L, Litauniece ZA, Bormane A, et al. Tick-borne encephalitis: A 43-year summary of epidemiological and clinical data from Latvia (1973 to 2016). PLoS One. 2018;13(11):e0204844.
